# Supplementary material for: Guidongnins I–J: Two New 6,7-seco-7,20-Olide-ent-kaurene Diterpenes with Unusual Structures from Isodon rubescens
Source: Int J Mol Sci. 2023 Aug 30;24(17):13451. doi: 10.3390/ijms241713451 (PMC10488066; doi:10.3390/ijms241713451)
Supplement: Supplementary file 1 [file ijms-24-13451-s001.zip › ijms-2551486-supplementary.pdf]

Supporting Information

Guidongnins I-J, two new 6,7-*seco*-7,20-olide-*ent*-kaurene diterpenes with unusual structures from *Isodon rubescens*

Juan Zou[a‡], Jiang-Hai Ye[a‡], Chen-Liang Zhao[ab], Jing-Jie Zhang[a], Ya-Hua Liu[a], Lu-Tai Pan[a], Kang He[a*] and and Hong-Jie Zhang [b, *]

[a] *School of Pharmacy, Guizhou University of Traditional Chinese Medicine, 4 Dongqing road, Guiyang 550025, China*

[b] *School of Chinese Medicine, Hong Kong Baptist University, 7 Baptist University Road, Hong Kong SAR 999077, China.*

[*] Corresponding author. Kang He: Tel: +86-13608529029; Fax: +86-0851- 88308058; E-mail: zoujuan466@gzy.edu.cn. Hong-Jie Zhang: Tel: +852-34112956; Fax: +852-34112461; E-mail: zhanghj@hkbu.edu.hk.

†Juan Zou and Jiang-Hai Ye contributed equally to this work.

Table of Contents

General Experimental Procedures 3
Figure S1. The IR (KBr disc) spectrum of 1. 4
Figure S2. The HR-ESI-MS spectrum of 1. 5
Figure S4. ^{13}C NMR spectrum of 1 in CD_3OD (150 MHz). 8
Figure S5. DEPT spectrum of 1 in CD_3OD. 9
Figure S6. HSQC spectrum of 1 in CD_3OD. 10
Figure S7. HMBC spectrum of 1 in CD_3OD. 11
Figure S8. ^{1}H-^{1}H COSY spectrum of 1 in CD_3OD. 12
Figure S9. NOESY spectrum of 1 in CD_3OD. 13
Figure S10. The IR (KBr disc) spectrum of 2. 13
Figure S11. The HR-ESI-MS spectrum of 2. 14
Figure S12. ^{1}H NMR spectrum of 2 in $CDCl_3$ (600 MHz). 15
Figure S13. ^{13}C NMR spectrum of 2 in $CDCl_3$ (150 MHz). 16
Figure S14. DEPT spectrum of 2 in $CDCl_3$. 17
Figure S15. HSQC spectrum of 2 in $CDCl_3$. 18
Figure S16. HMBC spectrum of 2 in $CDCl_3$. 19
Figure S17. ^{1}H-^{1}H COSY spectrum of 2 in $CDCl_3$. 20
Figure S18. NOESY spectrum of 2 in $CDCl_3$. 21
Crystal Structure Report for Compound 1 22
Crystal Structure Report for Compound 2 47

General Experimental Procedures

An XRC-1 micro melting point apparatus (Sichuan Weitai, Chengdu, China) was used for the measurement of compounds' melting points. Optical rotations were determined on a Jasco P-1010 polarimeter (Perkin-Elmer, Waltham, United States). The IR spectra was recorded on a VECTOR22 infrared spectrometer (Bruker, Karlsruhe, Germany) using KBr pellets method. A Bruker Avance spectrometer (600 MHz, Bruker, Karlsruhe, Germany) was used for recording the NMR spectra. The HR-ESI-MS data was obtained on a Bruker Q-TOF mass spectrometer (Bruker, Karlsruhe, Germany). X-ray data were collected on a Bruker APEX-II CCD instrument equipped with Cu Kα radiation for 1 (Bruker, Rheinstetten, Germany) and Bruker APEX-II instrument equipped with Mo Kα radiation for 2 (Bruker, Rheinstetten, Germany). Silica gel (200-300 mesh; Qingdao Haiyang Chemical, Qingdao, China) was used for column chromatography, while silica gel GF254 (0.2 mm, Qingdao Haiyang Chemical Inc., Qingdao, China) was used for analytical TLC. Prior to use, all solvents were distilled.

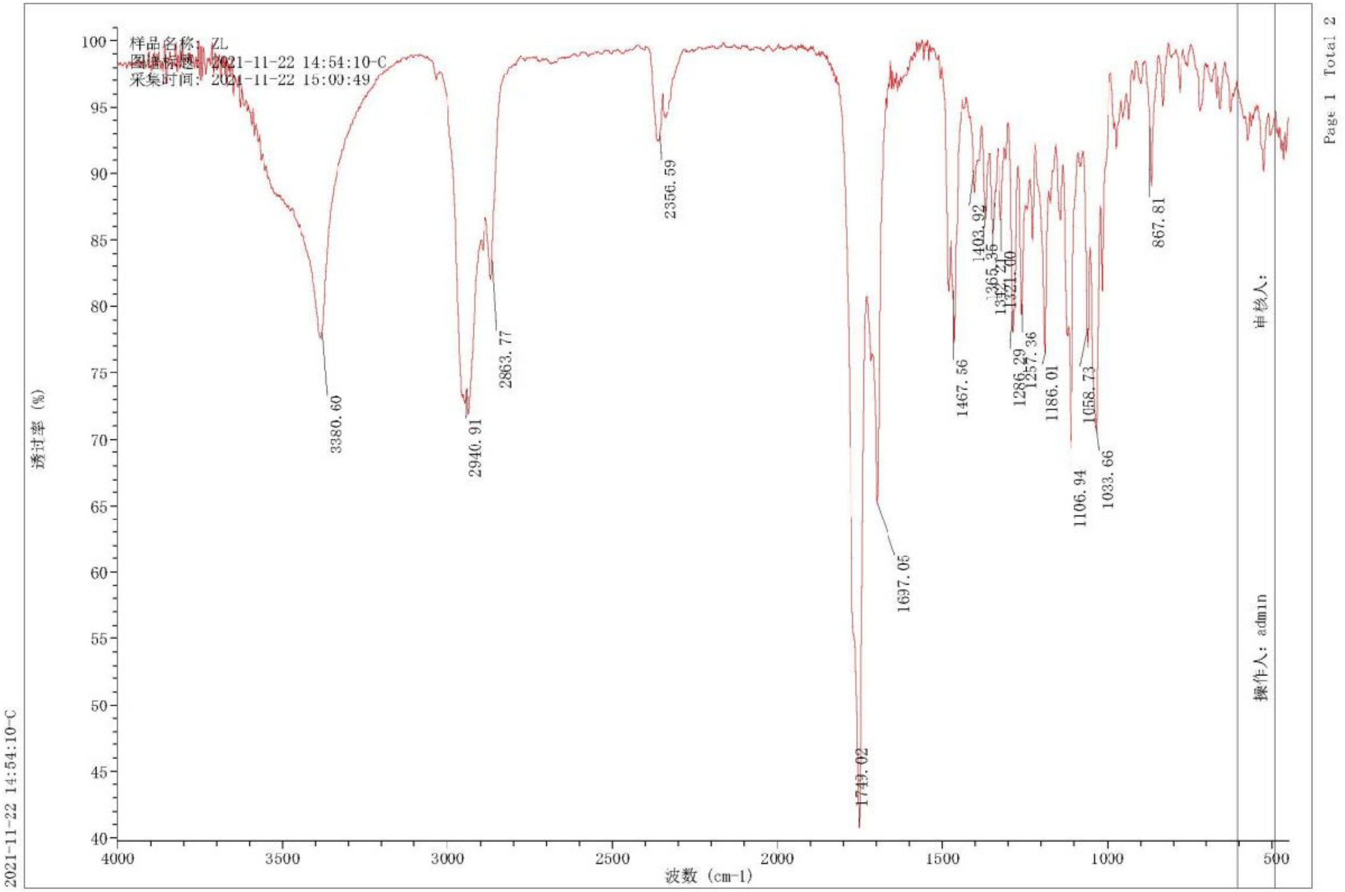

Figure S1. The IR (KBr disc) spectrum of **1.**

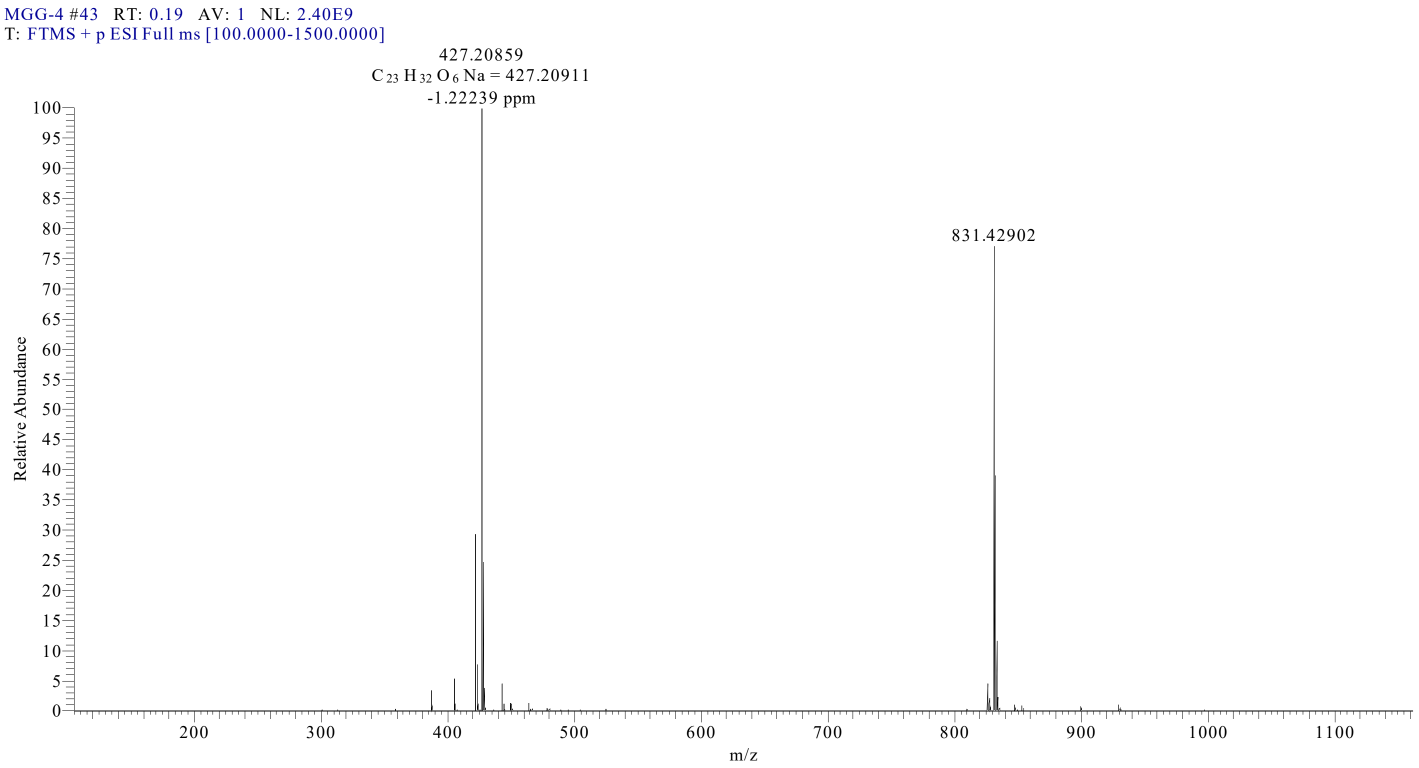

Figure S2. The HR-ESI-MS spectrum of **1.**

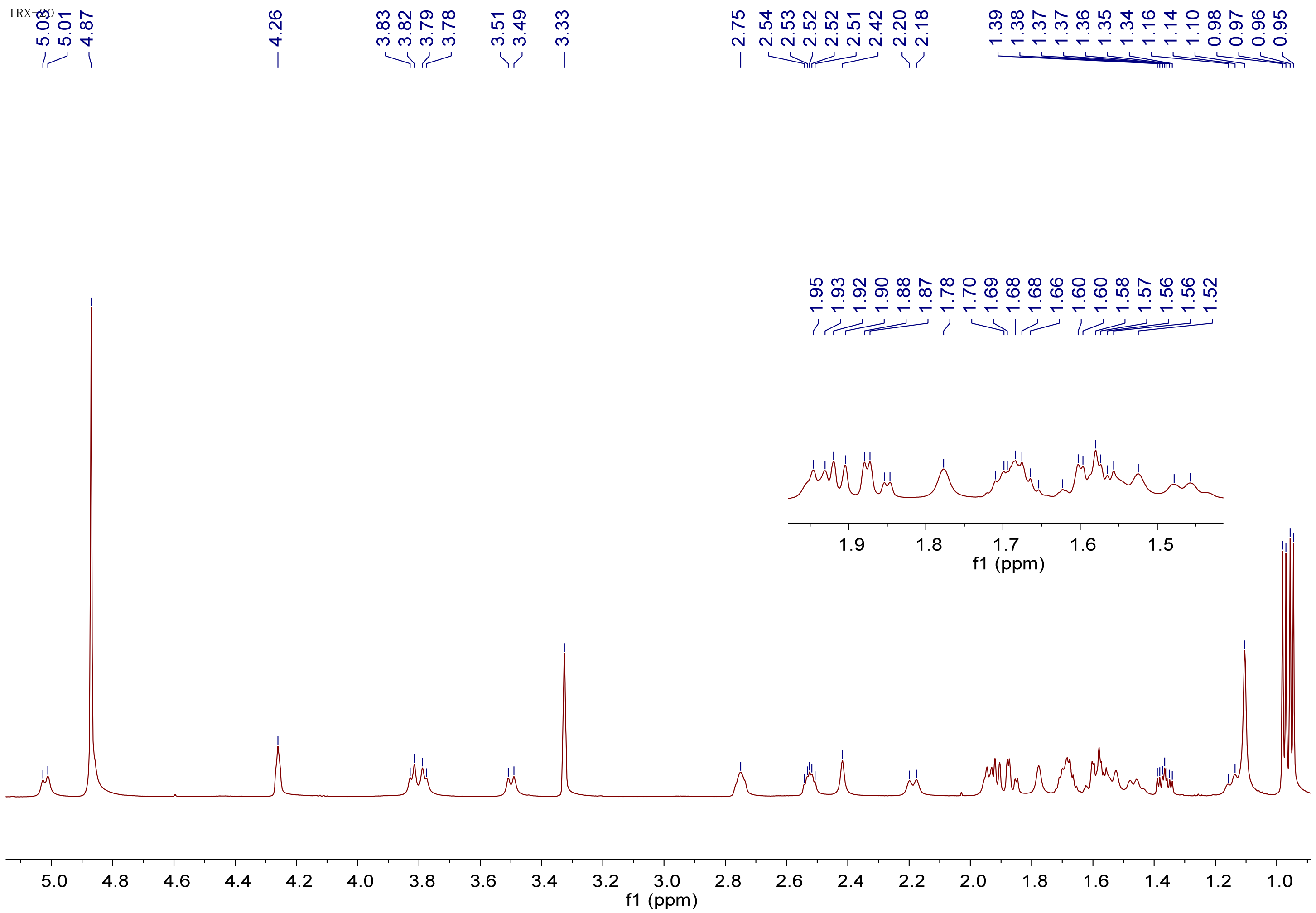

Figure S3. ^{1}H NMR spectrum of **1** in CD_3OD (600 MHz).

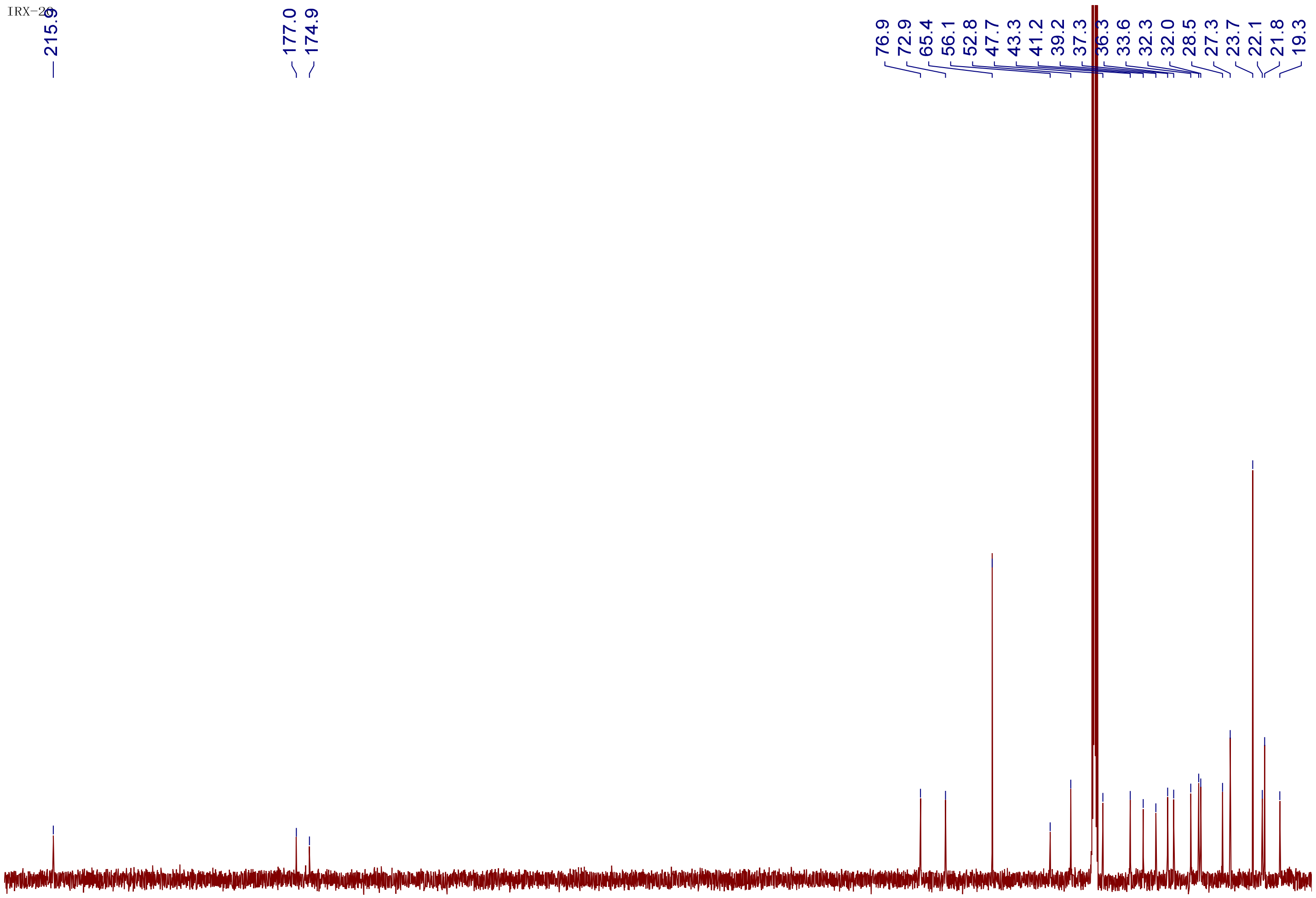
IRX-20
215.9
177.0
174.9
76.9
72.9
65.4
56.1
52.8
47.7
43.3
41.2
39.2
37.3
36.3
33.6
32.3
32.0
28.5
27.3
23.7
22.1
21.8
19.3

Figure S4. ^{13}C NMR spectrum of **1** in CD_3OD (150 MHz).

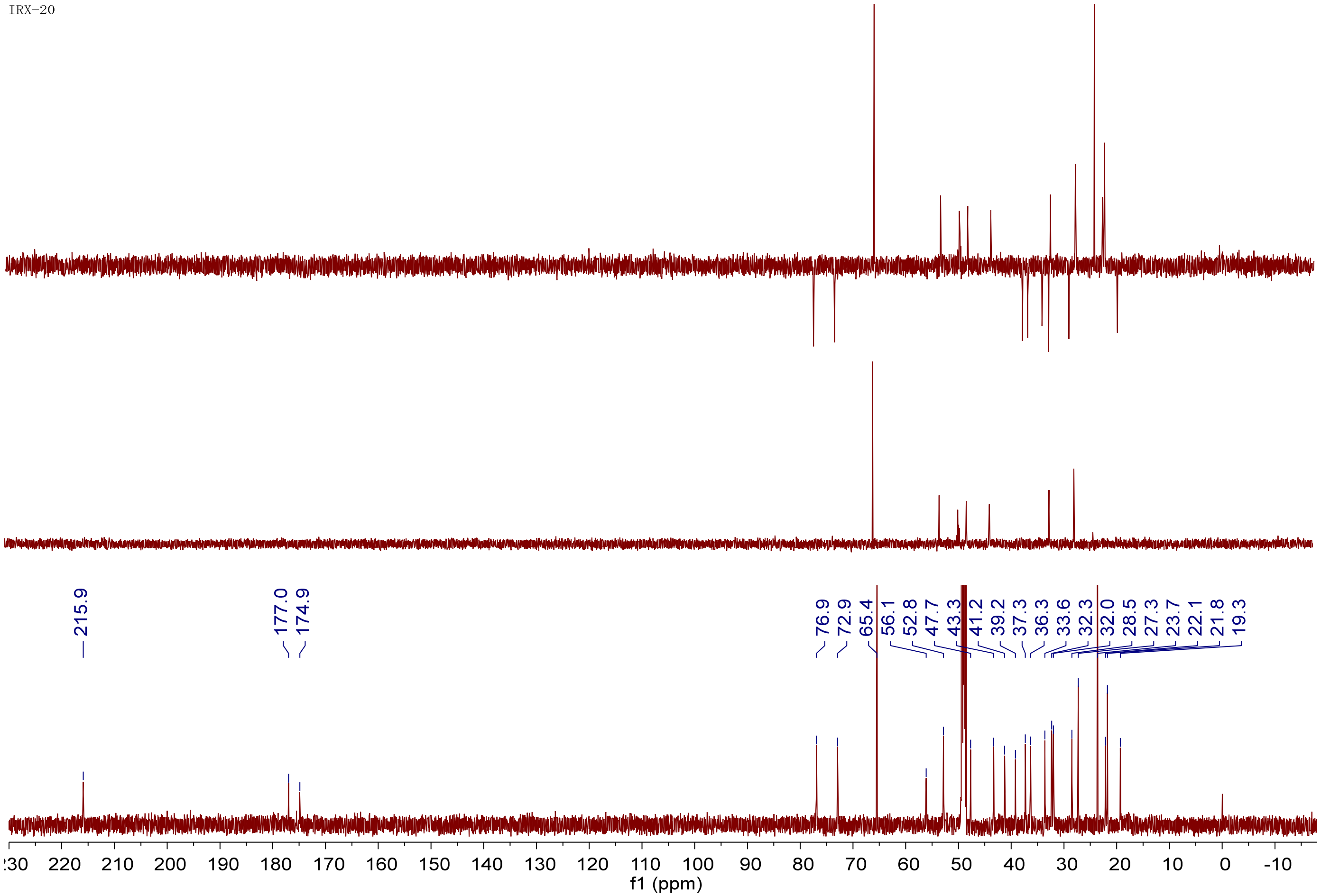

Figure S5. DEPT spectrum of **1** in CD_3OD.

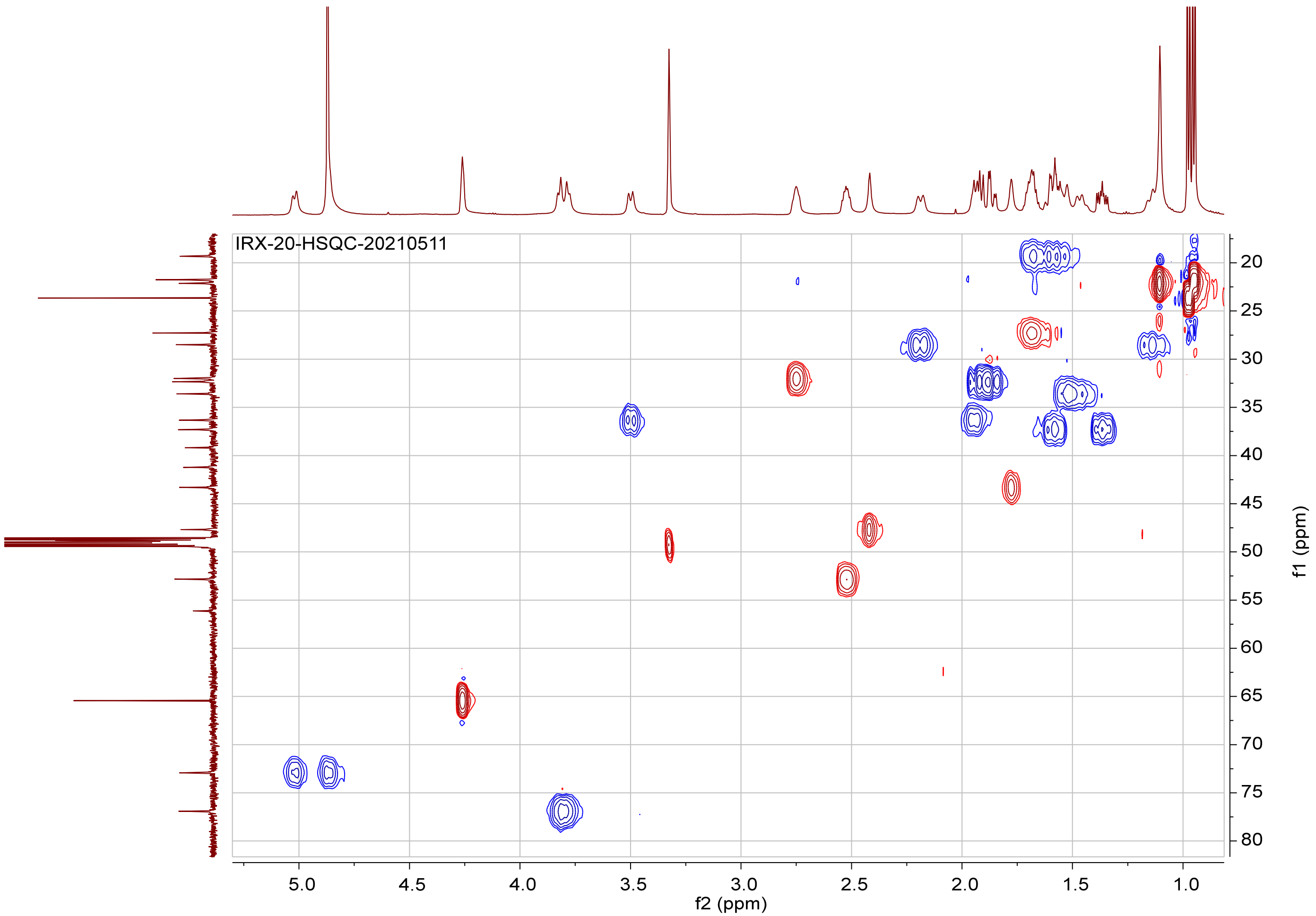

Figure S6. HSQC spectrum of **1** in CD_3OD.

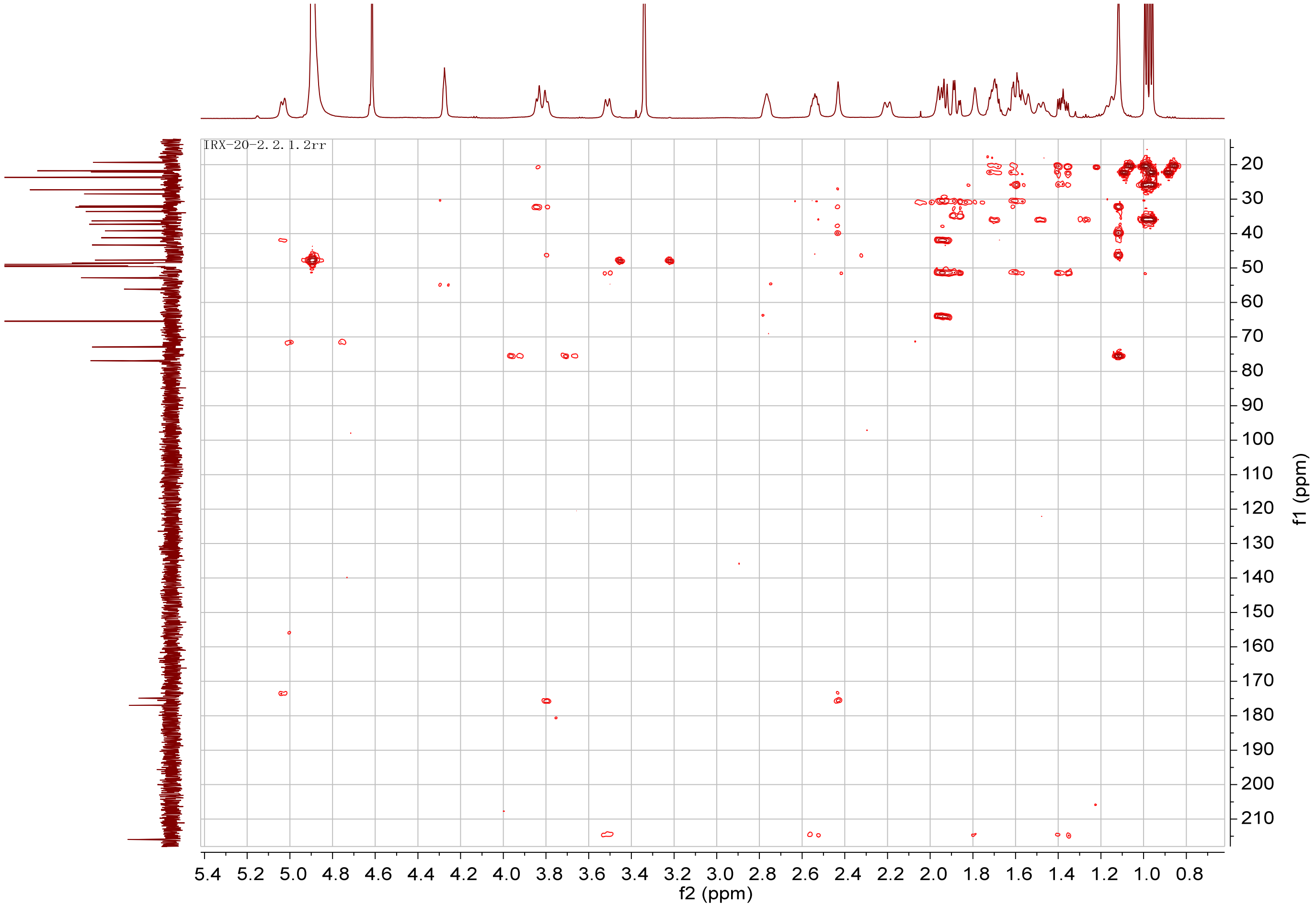

Figure S7. HMBC spectrum of **1** in CD_3OD.

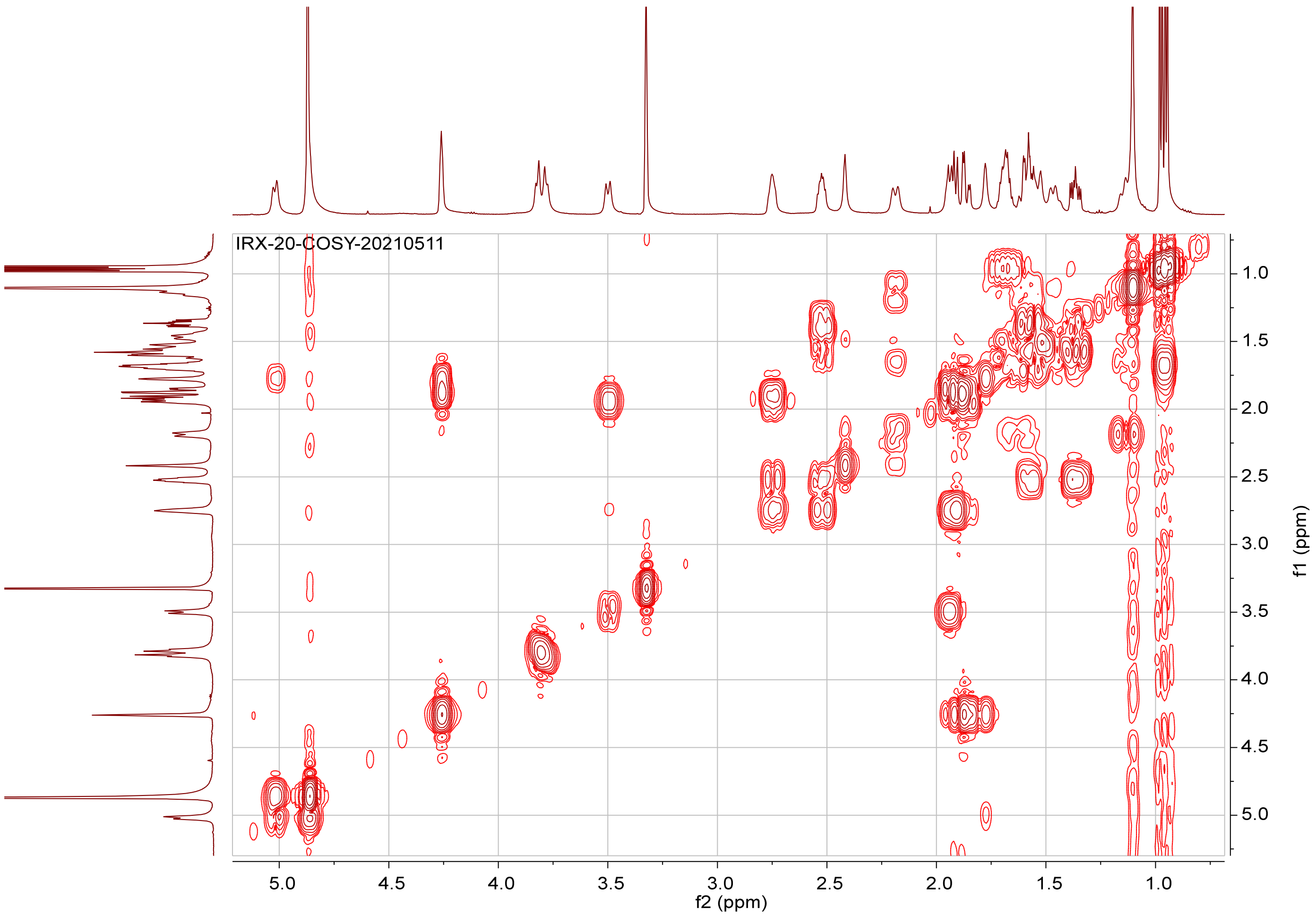

Figure S8. ^{1}H-^{1}H COSY spectrum of **1** in CD_3OD.

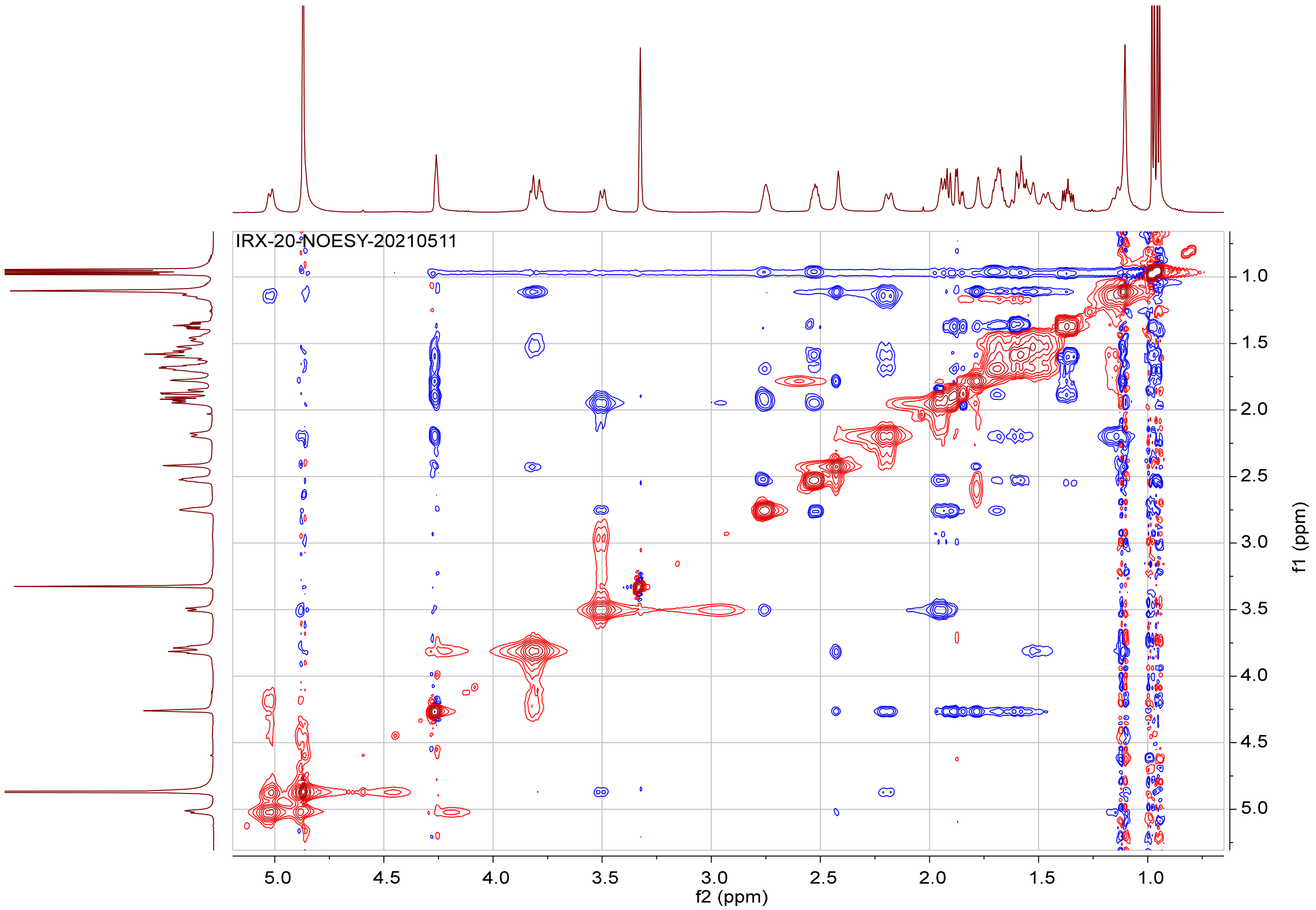

Figure S9. NOESY spectrum of **1** in CD_3OD.

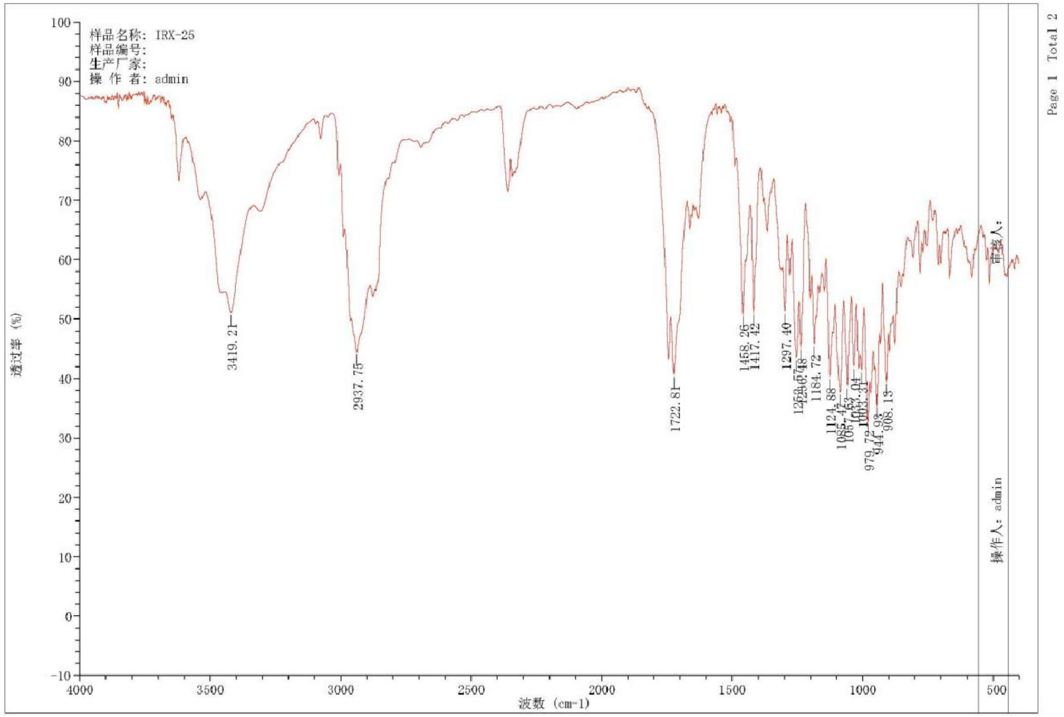

Figure S10. The IR (KBr disc) spectrum of **2.**

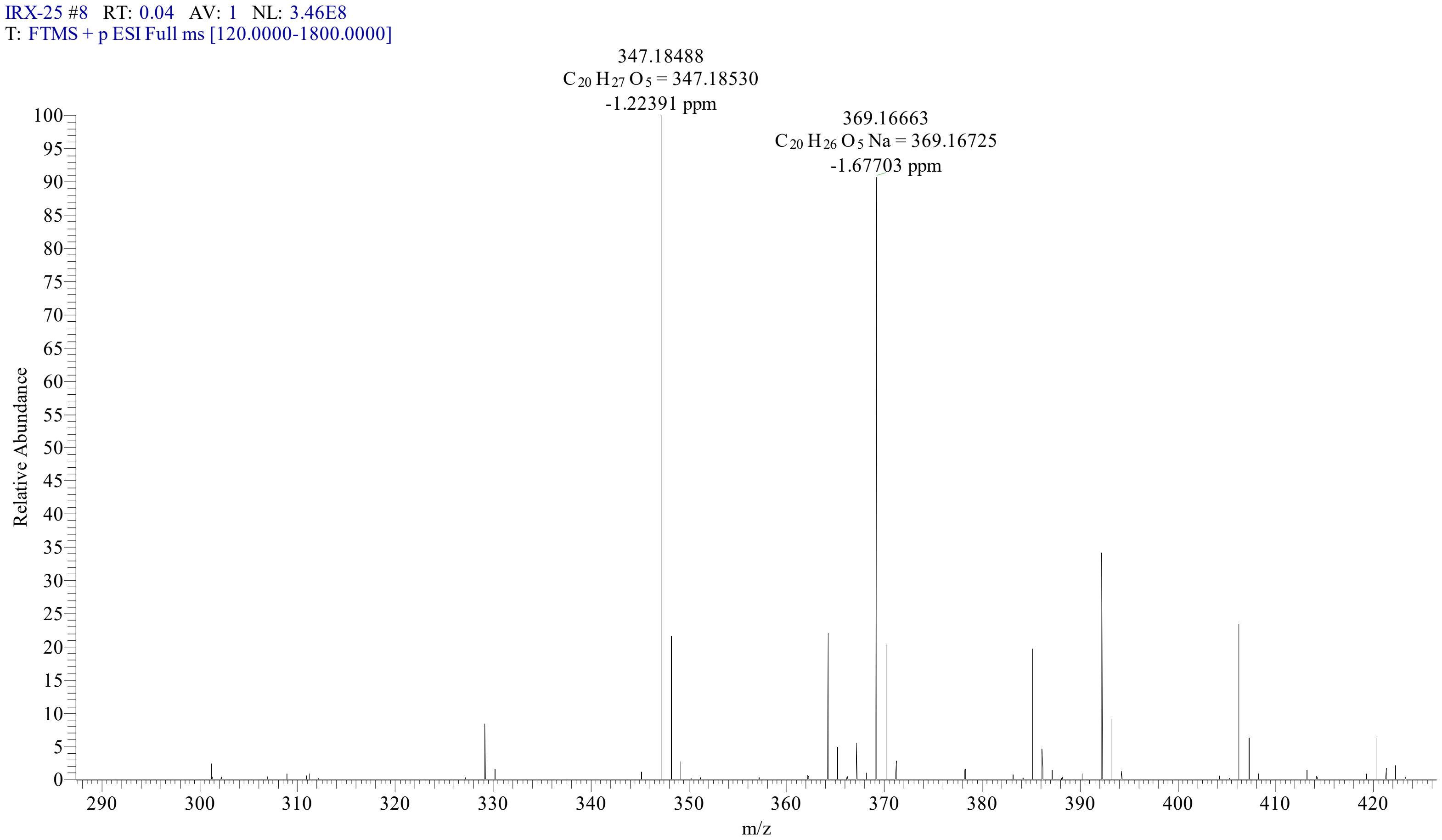

Figure S11. The HR-ESI-MS spectrum of **2**.

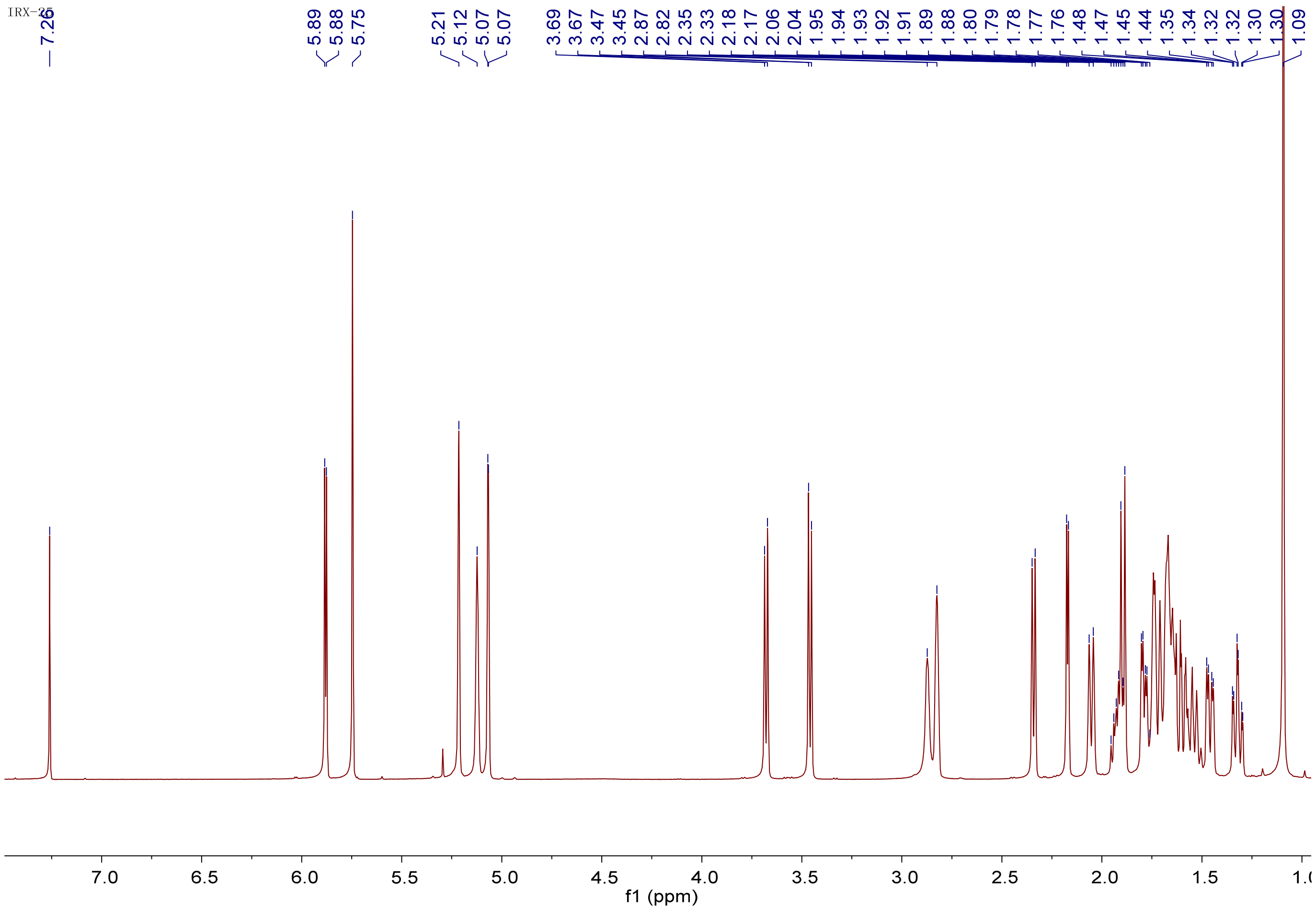

Figure S12. ^{1}H NMR spectrum of **2** in $CDCl_3$ (600 MHz).

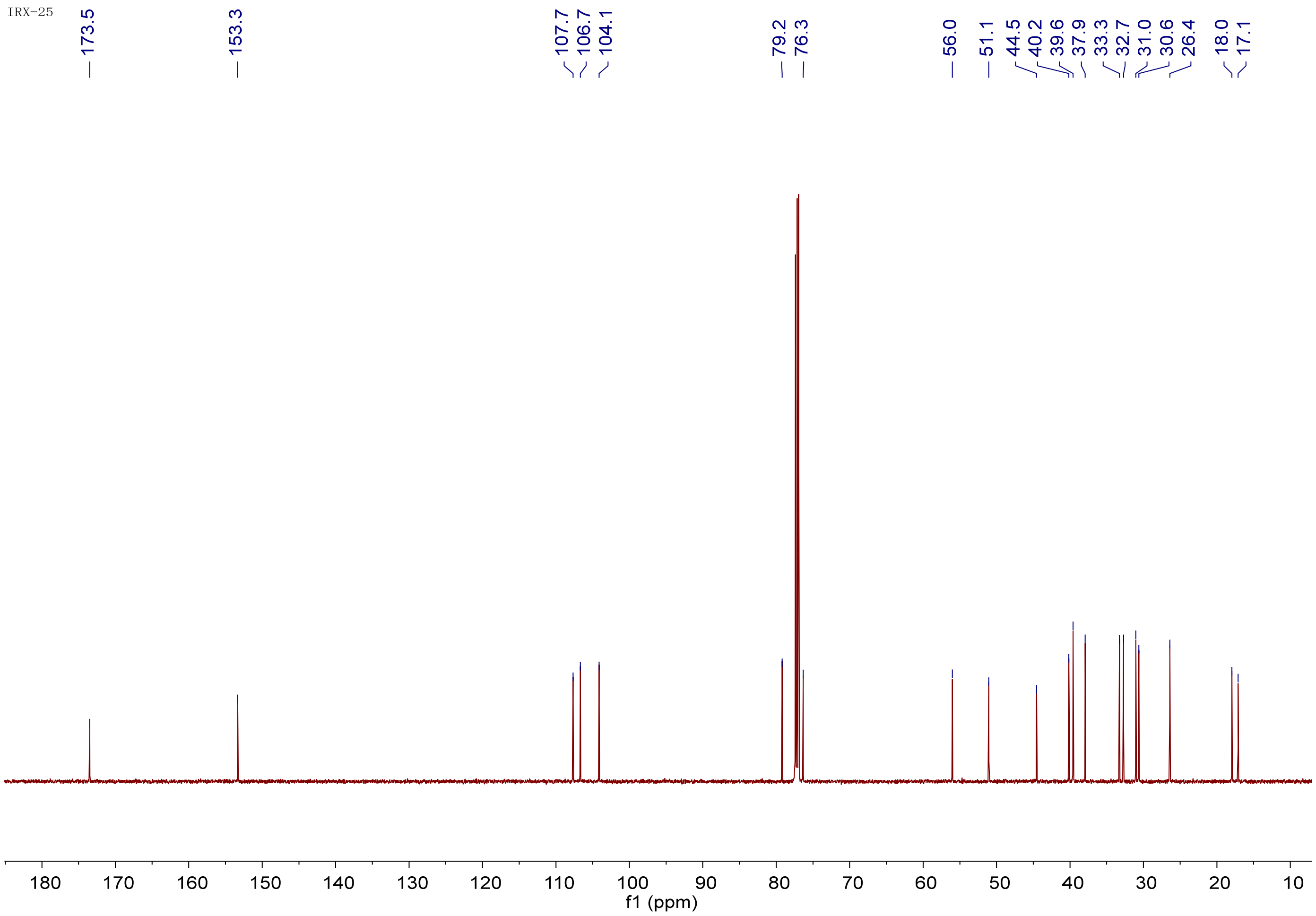

Figure S13. ^{13}C NMR spectrum of **2** in $CDCl_3$ (150 MHz).

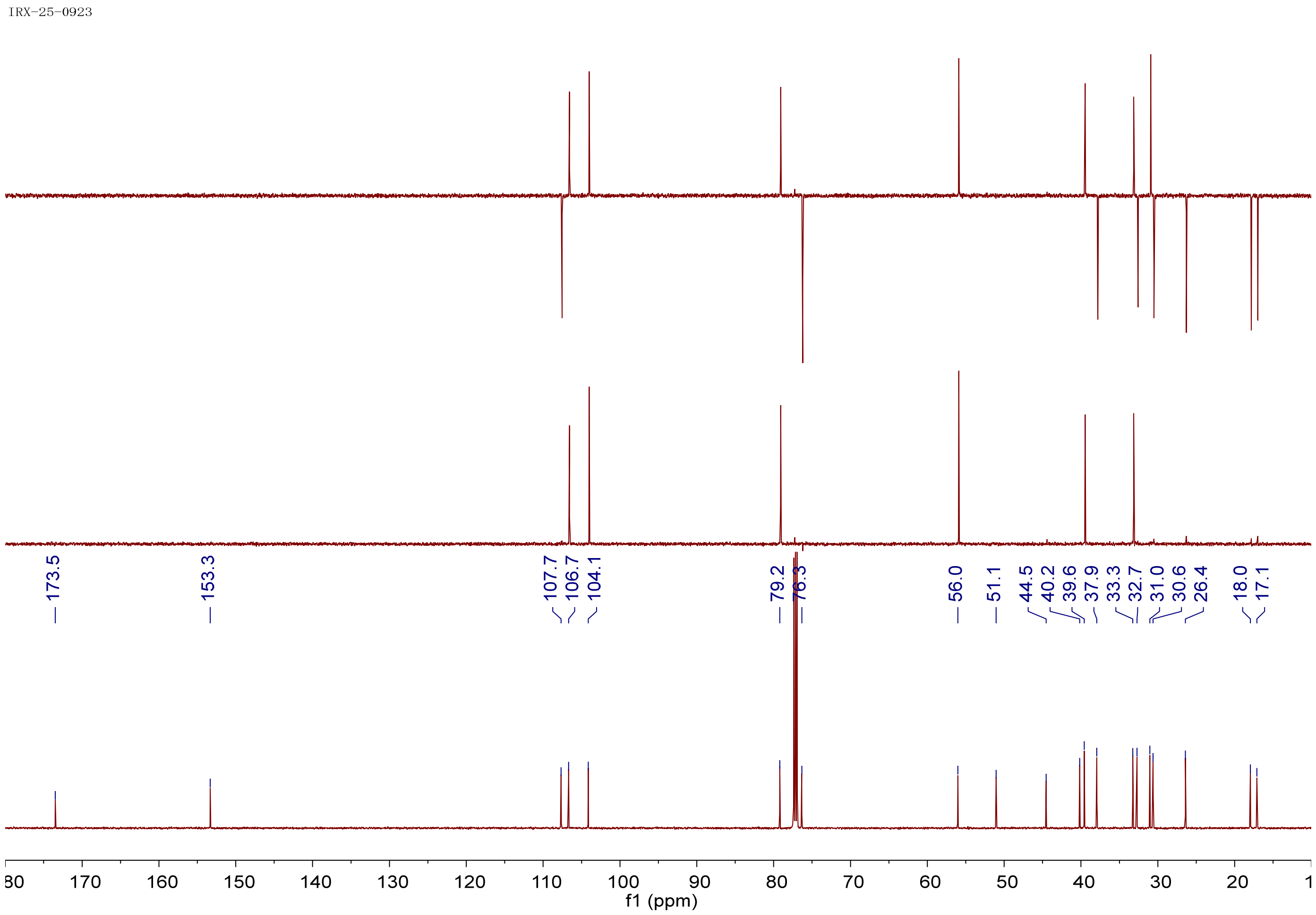

Figure S14. DEPT spectrum of **2** in $CDCl_3$.

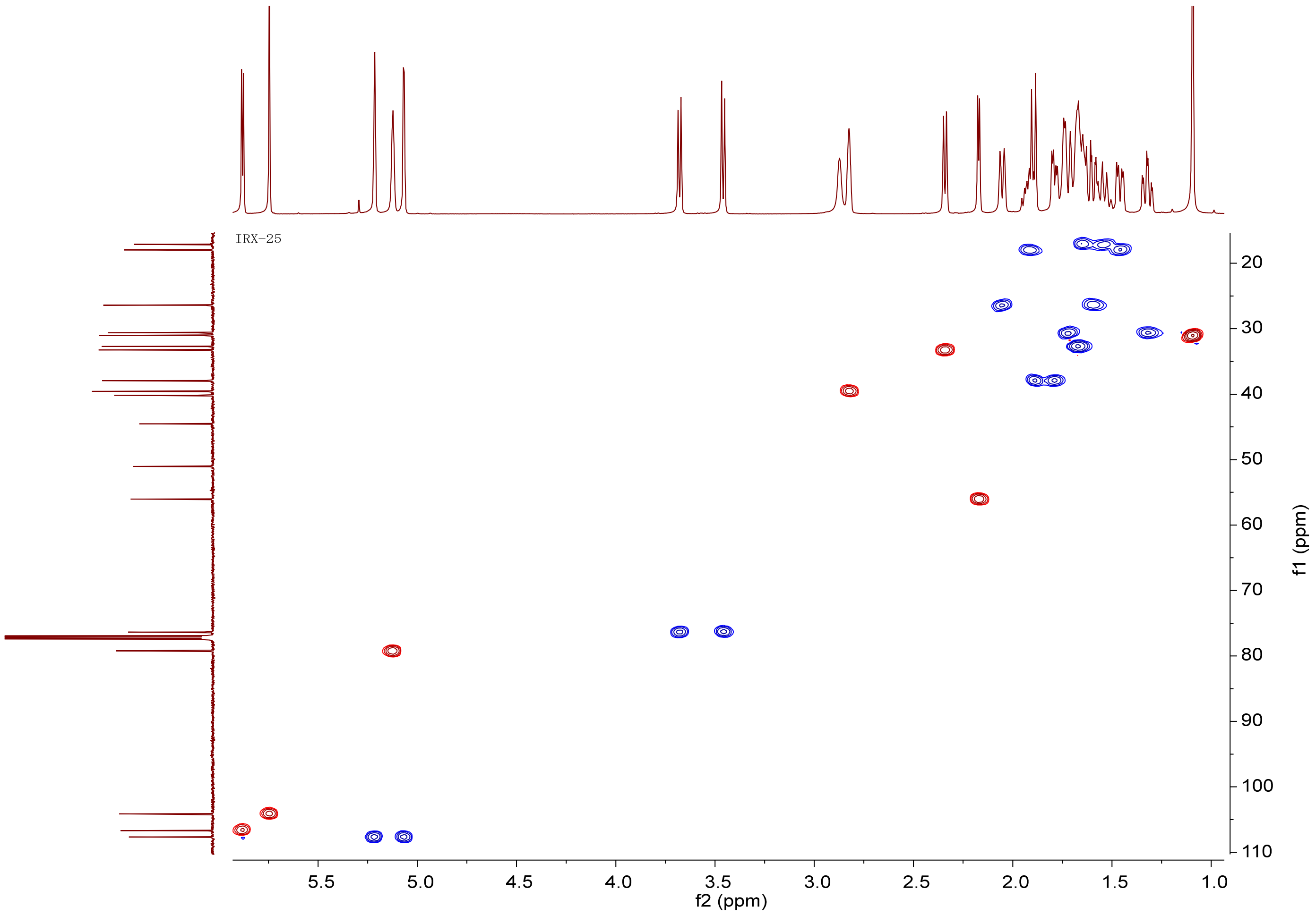

Figure S15. HSQC spectrum of **2** in $CDCl_3$.

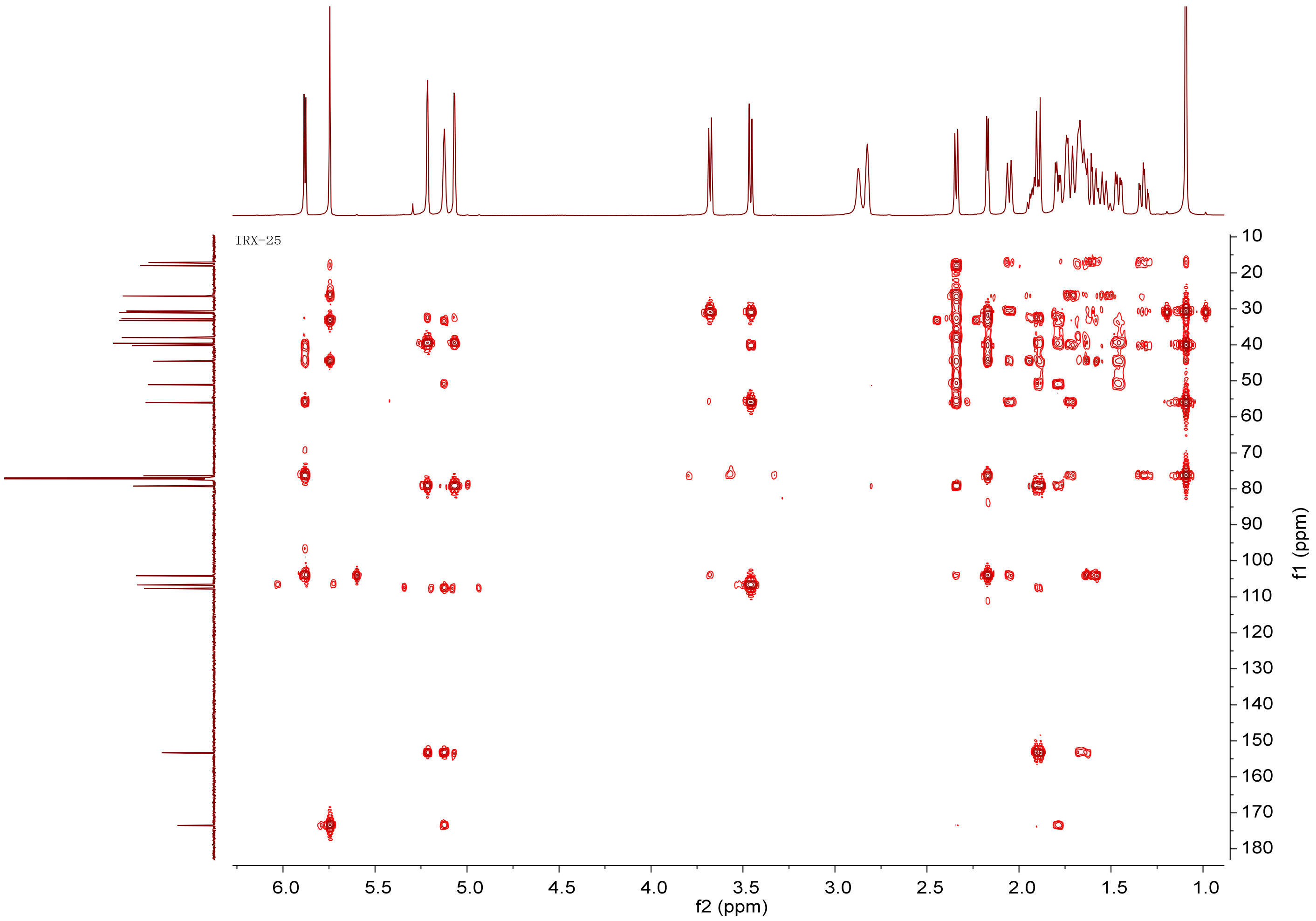

Figure S16. HMBC spectrum of **2** in $CDCl_3$.

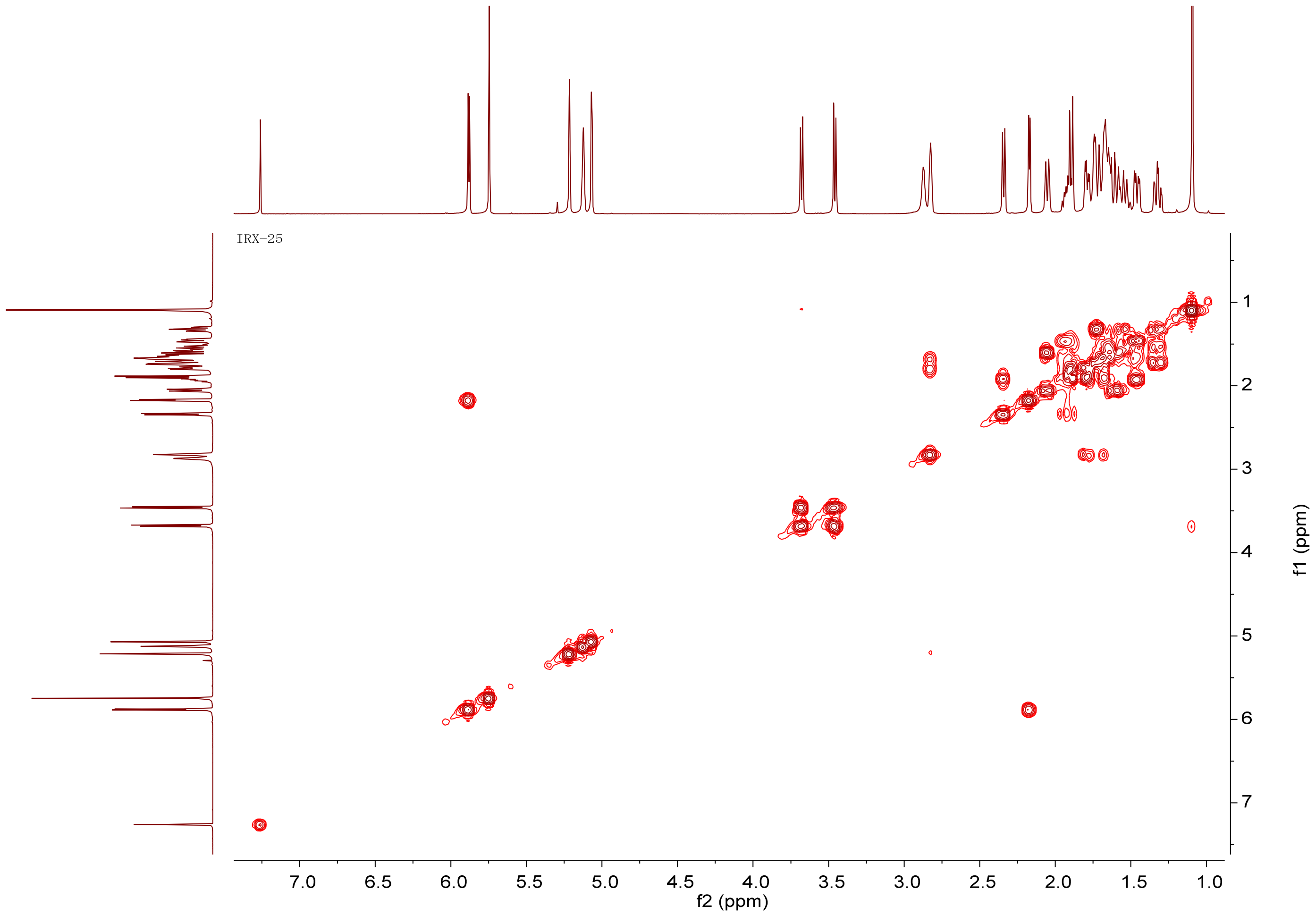

Figure S17. ^{1}H-^{1}H COSY spectrum of **2** in $CDCl_3$.

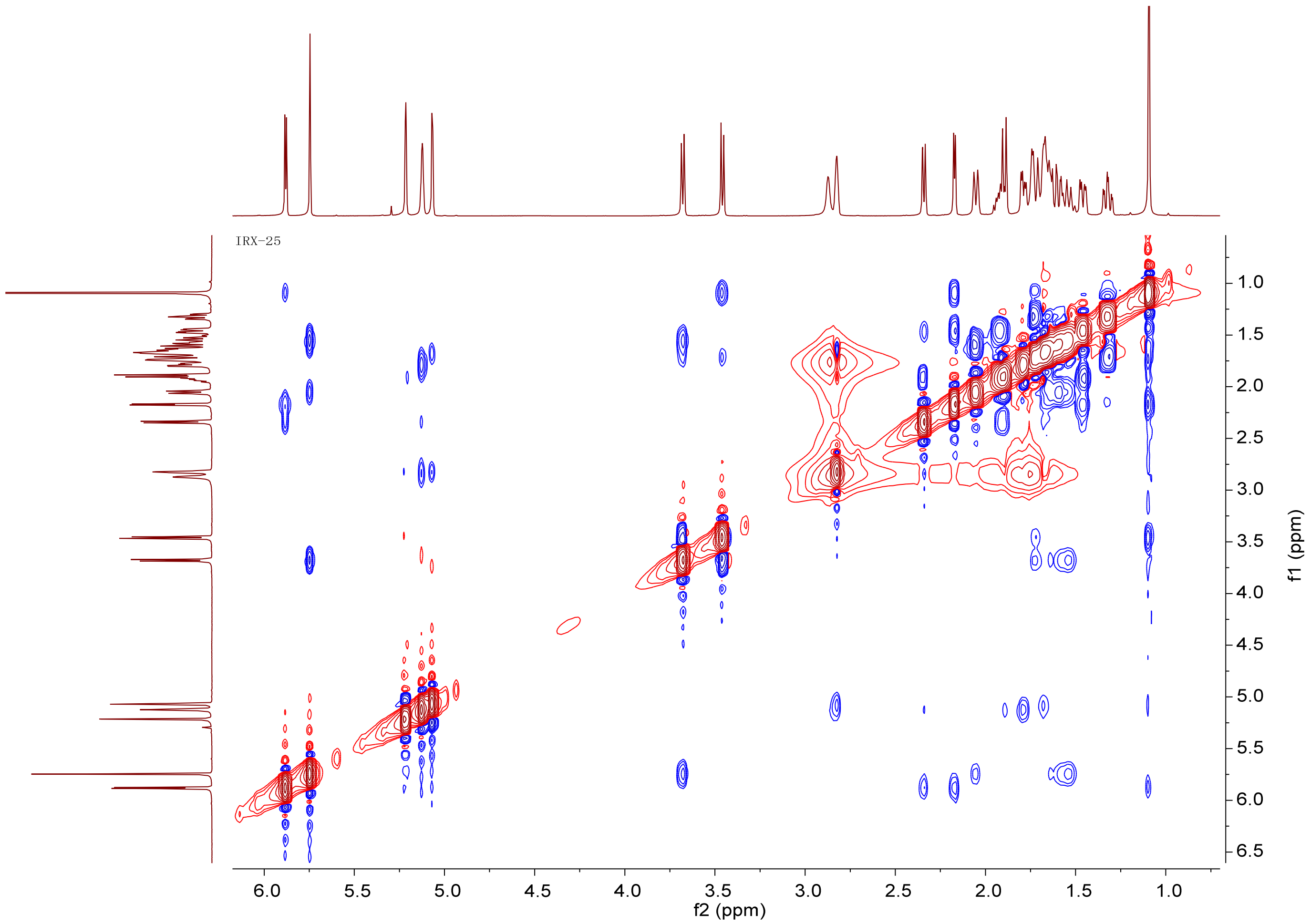

Figure S18. NOESY spectrum of **2** in $CDCl_3$.

Crystal Structure Report for Compound **1**

```
data_a
_audit_creation_date              2023-08-14
_audit_creation_method
;
Olex2 1.2-beta
(compiled 2018.05.29 svn.r3508 for OlexSys, GUI svn.r5506)
;
_shelx_SHELXL_version_number      '2014/7'
_audit_contact_author_address     ?
_audit_contact_author_email       ?
_audit_contact_author_name        ''
_audit_contact_author_phone       ?
_publ_contact_author_id_orcid     ?
_publ_section_references
;
Dolomanov, O.V., Bourhis, L.J., Gildea, R.J, Howard, J.A.K. & Puschmann, H.
 (2009), J. Appl. Cryst. 42, 339-341.

Sheldrick, G.M. (2015). Acta Cryst. A71, 3-8.

Sheldrick, G.M. (2015). Acta Cryst. C71, 3-8.
;
_chemical_name_common             ?
_chemical_name_systematic         ?
_chemical_formula_moiety          'C23 H32 O6'
```

```
_chemical_formula_sum                'C23 H32 O6'
_chemical_formula_weight             404.48
_chemical_absolute_configuration     ad
_chemical_melting_point              ?
loop_
    _atom_type_symbol
    _atom_type_description
    _atom_type_scat_dispersion_real
    _atom_type_scat_dispersion_imag
    _atom_type_scat_source
  'C' 'C' 0.0181 0.0091 'International Tables Vol C Tables 4.2.6.8 and 6.1.1.4'
  'H' 'H' 0.0000 0.0000 'International Tables Vol C Tables 4.2.6.8 and 6.1.1.4'
  'O' 'O' 0.0492 0.0322 'International Tables Vol C Tables 4.2.6.8 and 6.1.1.4'

_shelx_space_group_comment
;
The symmetry employed for this shelxl refinement is uniquely defined
by the following loop, which should always be used as a source of
symmetry information in preference to the above space-group names.
They are only intended as comments.
;
_space_group_crystal_system          'orthorhombic'
_space_group_IT_number               19
_space_group_name_H-M_alt            'P 21 21 21'
_space_group_name_Hall               'P 2ac 2ab'
loop_
    _space_group_symop_operation_xyz
  'x, y, z'
  '-x+1/2, -y, z+1/2'
```

```
 '-x, y+1/2, -z+1/2'
 'x+1/2, -y+1/2, -z'

_cell_length_a                    8.0160(4)
_cell_length_b                    13.0903(6)
_cell_length_c                    19.9957(9)
_cell_angle_alpha                 90
_cell_angle_beta                  90
_cell_angle_gamma                 90
_cell_volume                      2098.19(17)
_cell_formula_units_Z             4
_cell_measurement_reflns_used     9820
_cell_measurement_temperature     273(2)
_cell_measurement_theta_max       79.42
_cell_measurement_theta_min       4.04
_shelx_estimated_absorpt_T_max    0.896
_shelx_estimated_absorpt_T_min    0.877
_exptl_absorpt_coefficient_mu     0.746
_exptl_absorpt_correction_T_max   ?
_exptl_absorpt_correction_T_min   ?
_exptl_absorpt_correction_type    'none'
_exptl_absorpt_process_details    ?
_exptl_absorpt_special_details    ?
_exptl_crystal_colour             colourless
_exptl_crystal_density_diffrn     1.280
_exptl_crystal_density_meas       ?
_exptl_crystal_density_method     ?
_exptl_crystal_description        block
_exptl_crystal_F_000              872
```

_exptl_crystal_size_max 0.18
_exptl_crystal_size_mid 0.17
_exptl_crystal_size_min 0.15
_exptl_transmission_factor_max ?
_exptl_transmission_factor_min ?
_diffrn_reflns_av_R_equivalents 0.0688
_diffrn_reflns_av_unetI/netI 0.0359
_diffrn_reflns_Laue_measured_fraction_full 0.999
_diffrn_reflns_Laue_measured_fraction_max 0.994
_diffrn_reflns_limit_h_max 10
_diffrn_reflns_limit_h_min -9
_diffrn_reflns_limit_k_max 12
_diffrn_reflns_limit_k_min -16
_diffrn_reflns_limit_l_max 24
_diffrn_reflns_limit_l_min -25
_diffrn_reflns_number 30644
_diffrn_reflns_point_group_measured_fraction_full 0.999
_diffrn_reflns_point_group_measured_fraction_max 0.985
_diffrn_reflns_theta_full 67.679
_diffrn_reflns_theta_max 80.009
_diffrn_reflns_theta_min 4.036
_diffrn_ambient_temperature 273.15
_diffrn_detector_area_resol_mean ?
_diffrn_measured_fraction_theta_full 0.999
_diffrn_measured_fraction_theta_max 0.994
_diffrn_measurement_device_type 'Bruker APEX-II CCD'
_diffrn_measurement_method '\f and \w scans'
_diffrn_radiation_type CuK\a
_diffrn_radiation_wavelength 1.54178

```
_diffrn_source                        ?
_diffrn_standards_number              0
_reflns_Friedel_coverage              0.735
_reflns_Friedel_fraction_full         0.998
_reflns_Friedel_fraction_max          0.972
_reflns_number_gt                     4267
_reflns_number_total                  4524
_reflns_special_details
;
 Reflections were merged by SHELXL according to the crystal
 class for the calculation of statistics and refinement.

 _reflns_Friedel_fraction is defined as the number of unique
 Friedel pairs measured divided by the number that would be
 possible theoretically, ignoring centric projections and
 systematic absences.
;
_reflns_threshold_expression          'I > 2\s(I)'
_computing_cell_refinement            ?
_computing_data_collection            ?
_computing_data_reduction             ?
_computing_molecular_graphics         'Olex2 (Dolomanov et al., 2009)'
_computing_publication_material       'Olex2 (Dolomanov et al., 2009)'
_computing_structure_refinement       'ShelXL (Sheldrick, 2015)'
_computing_structure_solution         'ShelXT (Sheldrick, 2015)'
_refine_diff_density_max              0.336
_refine_diff_density_min              -0.262
_refine_diff_density_rms              0.053
_refine_ls_abs_structure_details
```

;
  Flack x determined using 1706 quotients [(I+)-(I-)]/[(I+)+(I-)]
  (Parsons, Flack and Wagner, Acta Cryst. B69 (2013) 249-259).
;
_refine_ls_abs_structure_Flack 0.00(6)
_refine_ls_extinction_coef .
_refine_ls_extinction_method none
_refine_ls_goodness_of_fit_ref 1.129
_refine_ls_hydrogen_treatment constr
_refine_ls_matrix_type full
_refine_ls_number_parameters 266
_refine_ls_number_reflns 4524
_refine_ls_number_restraints 0
_refine_ls_R_factor_all 0.0479
_refine_ls_R_factor_gt 0.0451
_refine_ls_restrained_S_all 1.129
_refine_ls_shift/su_max 0.000
_refine_ls_shift/su_mean 0.000
_refine_ls_structure_factor_coef Fsqd
_refine_ls_weighting_details
  'w=1/[\s^2^(Fo^2^)+(0.1000P)^2^] where P=(Fo^2^+2Fc^2^)/3'
_refine_ls_weighting_scheme calc
_refine_ls_wR_factor_gt 0.1309
_refine_ls_wR_factor_ref 0.1351
_refine_special_details ?
_olex2_refinement_description
;
1. Fixed Uiso
  At 1.2 times of:

```
    All C(H) groups, All C(H,H) groups
  At 1.5 times of:
    All C(H,H,H) groups, All O(H) groups
2.a Ternary CH refined with riding coordinates:
  C9(H9), C16(H16), C11(H11), C5(H5), C13(H13), C21(H21)
2.b Secondary CH2 refined with riding coordinates:
  C20(H20A,H20B), C14(H14A,H14B), C12(H12A,H12B), C1(H1A,H1B), C17(H17A,H17B),
  C3(H3A,H3B), C2(H2A,H2B), C19(H19A,H19B)
2.c Idealised Me refined as rotating group:
  C18(H18A,H18B,H18C), C23(H23A,H23B,H23C), C22(H22A,H22B,H22C)
2.d Idealised tetrahedral OH refined as rotating group:
  O1(H1)
;
_atom_sites_solution_hydrogens        geom
_atom_sites_solution_primary          dual
_atom_sites_solution_secondary        ?
loop_
    _atom_site_label
    _atom_site_type_symbol
    _atom_site_fract_x
    _atom_site_fract_y
    _atom_site_fract_z
    _atom_site_U_iso_or_equiv
    _atom_site_adp_type
    _atom_site_occupancy
    _atom_site_site_symmetry_order
    _atom_site_calc_flag
    _atom_site_refinement_flags_posn
    _atom_site_refinement_flags_adp
```

_atom_site_refinement_flags_occupancy
_atom_site_disorder_assembly
_atom_site_disorder_group
O3 O 0.0753(2) 0.54872(10) 0.67680(8) 0.0444(4) Uani 1 1 d . . . . .
O1 O -0.0413(2) 0.32034(11) 0.78771(10) 0.0481(4) Uani 1 1 d . . . . .
H1 H -0.0992 0.2689 0.7848 0.072 Uiso 1 1 calc GR . . . .
O6 O 0.5341(2) 0.52010(12) 0.75551(8) 0.0482(4) Uani 1 1 d . . . . .
O5 O 0.2242(3) 0.64999(10) 0.73988(9) 0.0555(5) Uani 1 1 d . . . . .
O4 O 0.3947(4) 0.42447(18) 0.50686(9) 0.0731(7) Uani 1 1 d . . . . .
O2 O 0.1331(3) 0.46695(19) 0.52593(10) 0.0746(7) Uani 1 1 d . . . . .
C9 C 0.2257(2) 0.36435(11) 0.73769(8) 0.0281(4) Uani 1 1 d . . . . .
H9 H 0.3400 0.3380 0.7356 0.034 Uiso 1 1 calc R . . . .
C15 C 0.4235(3) 0.48450(12) 0.78914(9) 0.0340(4) Uani 1 1 d . . . . .
C10 C 0.1637(3) 0.36921(12) 0.66416(9) 0.0313(4) Uani 1 1 d . . . . .
C16 C 0.4414(3) 0.43742(13) 0.85878(9) 0.0363(4) Uani 1 1 d . . . . .
H16 H 0.4711 0.4933 0.8891 0.044 Uiso 1 1 calc R . . . .
C20 C 0.0188(3) 0.44506(13) 0.66282(11) 0.0408(5) Uani 1 1 d . . . . .
H20A H -0.0339 0.4432 0.6192 0.049 Uiso 1 1 calc R . . . .
H20B H -0.0636 0.4251 0.6958 0.049 Uiso 1 1 calc R . . . .
C11 C 0.1306(3) 0.29228(12) 0.78524(10) 0.0363(4) Uani 1 1 d . . . . .
H11 H 0.1392 0.2224 0.7680 0.044 Uiso 1 1 calc R . . . .
C8 C 0.2397(3) 0.47478(12) 0.76891(9) 0.0314(4) Uani 1 1 d . . . . .
C14 C 0.1567(3) 0.48215(14) 0.83884(10) 0.0396(4) Uani 1 1 d . . . . .
H14A H 0.1668 0.5502 0.8576 0.048 Uiso 1 1 calc R . . . .
H14B H 0.0400 0.4626 0.8374 0.048 Uiso 1 1 calc R . . . .
C7 C 0.1819(3) 0.56352(13) 0.72668(10) 0.0382(5) Uani 1 1 d . . . . .
C5 C 0.3105(3) 0.40420(12) 0.61881(9) 0.0340(4) Uani 1 1 d . . . . .
H5 H 0.3627 0.4635 0.6403 0.041 Uiso 1 1 calc R . . . .
C12 C 0.2099(3) 0.29591(15) 0.85515(10) 0.0430(5) Uani 1 1 d . . . . .

H12A H 0.3076 0.2522 0.8555 0.052 Uiso 1 1 calc R . . . .
H12B H 0.1310 0.2686 0.8873 0.052 Uiso 1 1 calc R . . . .
C1 C 0.0993(3) 0.26567(14) 0.63804(10) 0.0396(5) Uani 1 1 d . . . . .
H1A H 0.0170 0.2391 0.6691 0.048 Uiso 1 1 calc R . . . .
H1B H 0.0445 0.2761 0.5953 0.048 Uiso 1 1 calc R . . . .
C17 C 0.5819(3) 0.35943(17) 0.86297(10) 0.0434(5) Uani 1 1 d . . . . .
H17A H 0.6833 0.3899 0.8456 0.052 Uiso 1 1 calc R . . . .
H17B H 0.5544 0.3015 0.8348 0.052 Uiso 1 1 calc R . . . .
C13 C 0.2617(3) 0.40442(14) 0.87738(9) 0.0384(4) Uani 1 1 d . . . . .
H13 H 0.2437 0.4125 0.9256 0.046 Uiso 1 1 calc R . . . .
C4 C 0.4502(3) 0.32587(15) 0.60369(10) 0.0419(5) Uani 1 1 d . . . . .
C21 C 0.6141(4) 0.32140(18) 0.93460(11) 0.0496(6) Uani 1 1 d . . . . .
H21 H 0.5060 0.3148 0.9571 0.059 Uiso 1 1 calc R . . . .
C3 C 0.3706(4) 0.22416(17) 0.58130(12) 0.0516(6) Uani 1 1 d . . . . .
H3A H 0.3211 0.2331 0.5374 0.062 Uiso 1 1 calc R . . . .
H3B H 0.4570 0.1726 0.5776 0.062 Uiso 1 1 calc R . . . .
C2 C 0.2380(4) 0.18703(14) 0.62946(12) 0.0485(6) Uani 1 1 d . . . . .
H2A H 0.2887 0.1733 0.6726 0.058 Uiso 1 1 calc R . . . .
H2B H 0.1908 0.1237 0.6129 0.058 Uiso 1 1 calc R . . . .
C6 C 0.2626(4) 0.43573(17) 0.54741(11) 0.0515(6) Uani 1 1 d . . . . .
C18 C 0.5841(4) 0.3081(2) 0.65691(13) 0.0549(6) Uani 1 1 d . . . . .
H18A H 0.6153 0.3723 0.6765 0.082 Uiso 1 1 calc GR . . . .
H18B H 0.6802 0.2771 0.6366 0.082 Uiso 1 1 calc GR . . . .
H18C H 0.5410 0.2638 0.6910 0.082 Uiso 1 1 calc GR . . . .
C19 C 0.5293(5) 0.3794(2) 0.54350(13) 0.0635(7) Uani 1 1 d . . . . .
H19A H 0.6072 0.4315 0.5582 0.076 Uiso 1 1 calc R . . . .
H19B H 0.5888 0.3306 0.5159 0.076 Uiso 1 1 calc R . . . .
C23 C 0.7188(6) 0.3976(3) 0.97398(16) 0.0776(10) Uani 1 1 d . . . . .
H23A H 0.8283 0.4018 0.9547 0.116 Uiso 1 1 calc GR . . . .

```
 H23B H 0.7275 0.3755 1.0196 0.116 Uiso 1 1 calc GR . . . .
 H23C H 0.6668 0.4636 0.9724 0.116 Uiso 1 1 calc GR . . . .
 C22 C 0.6962(6) 0.2169(2) 0.93384(16) 0.0803(11) Uani 1 1 d . . . . .
 H22A H 0.6244 0.1690 0.9116 0.120 Uiso 1 1 calc GR . . . .
 H22B H 0.7155 0.1947 0.9789 0.120 Uiso 1 1 calc GR . . . .
 H22C H 0.8006 0.2209 0.9105 0.120 Uiso 1 1 calc GR . . . .

loop_
   _atom_site_aniso_label
   _atom_site_aniso_U_11
   _atom_site_aniso_U_22
   _atom_site_aniso_U_33
   _atom_site_aniso_U_23
   _atom_site_aniso_U_13
   _atom_site_aniso_U_12
 O3 0.0497(10) 0.0295(6) 0.0539(8) 0.0016(5) -0.0142(8) 0.0097(6)
 O1 0.0374(9) 0.0405(7) 0.0663(10) -0.0033(6) 0.0083(8) -0.0097(6)
 O6 0.0469(9) 0.0519(8) 0.0458(8) 0.0088(6) -0.0030(7) -0.0132(7)
 O5 0.0756(14) 0.0252(6) 0.0656(10) -0.0003(6) -0.0199(10) 0.0031(7)
 O4 0.0939(18) 0.0898(13) 0.0354(8) 0.0162(9) 0.0101(10) 0.0041(13)
 O2 0.0833(17) 0.0896(13) 0.0509(10) 0.0249(9) -0.0196(11) 0.0100(13)
 C9 0.0337(10) 0.0223(6) 0.0282(7) 0.0001(5) -0.0012(7) -0.0011(6)
 C15 0.0418(12) 0.0280(7) 0.0322(8) -0.0019(6) -0.0033(8) -0.0011(7)
 C10 0.0334(10) 0.0286(7) 0.0320(8) -0.0006(6) -0.0053(7) -0.0008(7)
 C16 0.0443(12) 0.0355(8) 0.0289(8) -0.0018(6) -0.0035(8) 0.0025(8)
 C20 0.0378(12) 0.0349(8) 0.0496(10) -0.0004(7) -0.0128(9) 0.0034(8)
 C11 0.0426(12) 0.0273(7) 0.0389(9) 0.0025(6) 0.0050(9) -0.0042(7)
 C8 0.0393(11) 0.0236(6) 0.0313(8) -0.0012(6) -0.0015(8) 0.0007(7)
 C14 0.0455(12) 0.0357(7) 0.0377(9) -0.0065(7) 0.0035(9) 0.0055(8)
```

C7 0.0479(12) 0.0244(7) 0.0423(9) -0.0010(6) -0.0050(9) 0.0060(8)
C5 0.0409(12) 0.0329(7) 0.0281(7) 0.0031(6) -0.0037(7) -0.0016(7)
C12 0.0545(14) 0.0378(8) 0.0367(9) 0.0083(7) 0.0064(10) -0.0042(9)
C1 0.0429(12) 0.0355(8) 0.0404(9) -0.0067(7) -0.0071(9) -0.0067(8)
C17 0.0477(13) 0.0471(10) 0.0353(9) 0.0048(7) -0.0028(9) 0.0097(9)
C13 0.0481(12) 0.0399(8) 0.0272(8) -0.0021(6) 0.0046(8) 0.0024(8)
C4 0.0444(12) 0.0468(10) 0.0345(9) -0.0031(7) 0.0033(9) 0.0041(9)
C21 0.0526(15) 0.0576(11) 0.0385(10) 0.0112(8) -0.0010(10) 0.0070(11)
C3 0.0622(17) 0.0455(10) 0.0473(11) -0.0140(8) 0.0014(12) 0.0095(10)
C2 0.0618(15) 0.0298(7) 0.0537(11) -0.0094(7) -0.0028(11) 0.0007(9)
C6 0.0710(18) 0.0501(10) 0.0333(10) 0.0102(8) -0.0066(11) 0.0003(12)
C18 0.0408(14) 0.0717(14) 0.0523(12) -0.0025(11) 0.0007(10) 0.0132(13)
C19 0.0663(18) 0.0762(16) 0.0478(12) 0.0040(11) 0.0188(13) 0.0002(15)
C23 0.093(3) 0.0840(19) 0.0554(15) 0.0022(13) -0.0285(17) 0.0015(19)
C22 0.112(3) 0.0673(15) 0.0616(16) 0.0209(13) -0.0081(19) 0.0332(19)

_geom_special_details
;
All esds (except the esd in the dihedral angle between two l.s. planes)
are estimated using the full covariance matrix. The cell esds are taken
into account individually in the estimation of esds in distances, angles
and torsion angles; correlations between esds in cell parameters are only
used when they are defined by crystal symmetry. An approximate (isotropic)
treatment of cell esds is used for estimating esds involving l.s. planes.
;
loop_
_geom_bond_atom_site_label_1
_geom_bond_atom_site_label_2
_geom_bond_distance

_geom_bond_site_symmetry_2
_geom_bond_publ_flag
O3 C20 1.457(2) . ?
O3 C7 1.328(3) . ?
O1 H1 0.8200 . ?
O1 C11 1.427(3) . ?
O6 C15 1.206(3) . ?
O5 C7 1.211(2) . ?
O4 C6 1.342(4) . ?
O4 C19 1.432(4) . ?
O2 C6 1.195(4) . ?
C9 H9 0.9800 . ?
C9 C10 1.553(2) . ?
C9 C11 1.541(2) . ?
C9 C8 1.579(2) . ?
C15 C16 1.529(2) . ?
C15 C8 1.533(3) . ?
C10 C20 1.528(3) . ?
C10 C5 1.555(3) . ?
C10 C1 1.541(2) . ?
C16 H16 0.9800 . ?
C16 C17 1.523(3) . ?
C16 C13 1.549(3) . ?
C20 H20A 0.9700 . ?
C20 H20B 0.9700 . ?
C11 H11 0.9800 . ?
C11 C12 1.536(3) . ?
C8 C14 1.551(3) . ?
C8 C7 1.509(2) . ?

C14 H14A 0.9700 . ?
C14 H14B 0.9700 . ?
C14 C13 1.529(3) . ?
C5 H5 0.9800 . ?
C5 C4 1.548(3) . ?
C5 C6 1.535(3) . ?
C12 H12A 0.9700 . ?
C12 H12B 0.9700 . ?
C12 C13 1.545(3) . ?
C1 H1A 0.9700 . ?
C1 H1B 0.9700 . ?
C1 C2 1.525(3) . ?
C17 H17A 0.9700 . ?
C17 H17B 0.9700 . ?
C17 C21 1.538(3) . ?
C13 H13 0.9800 . ?
C4 C3 1.543(3) . ?
C4 C18 1.529(4) . ?
C4 C19 1.530(3) . ?
C21 H21 0.9800 . ?
C21 C23 1.523(5) . ?
C21 C22 1.518(4) . ?
C3 H3A 0.9700 . ?
C3 H3B 0.9700 . ?
C3 C2 1.514(4) . ?
C2 H2A 0.9700 . ?
C2 H2B 0.9700 . ?
C18 H18A 0.9600 . ?
C18 H18B 0.9600 . ?

C18 H18C 0.9600 . ?
C19 H19A 0.9700 . ?
C19 H19B 0.9700 . ?
C23 H23A 0.9600 . ?
C23 H23B 0.9600 . ?
C23 H23C 0.9600 . ?
C22 H22A 0.9600 . ?
C22 H22B 0.9600 . ?
C22 H22C 0.9600 . ?

loop_
 _geom_angle_atom_site_label_1
 _geom_angle_atom_site_label_2
 _geom_angle_atom_site_label_3
 _geom_angle
 _geom_angle_site_symmetry_1
 _geom_angle_site_symmetry_3
 _geom_angle_publ_flag
C7 O3 C20 118.67(15) . . ?
C11 O1 H1 109.5 . . ?
C6 O4 C19 109.33(19) . . ?
C10 C9 H9 105.8 . . ?
C10 C9 C8 111.07(12) . . ?
C11 C9 H9 105.8 . . ?
C11 C9 C10 116.81(15) . . ?
C11 C9 C8 110.59(14) . . ?
C8 C9 H9 105.8 . . ?
O6 C15 C16 126.5(2) . . ?
O6 C15 C8 126.26(18) . . ?

C16 C15 C8 107.26(17) . . ?
C9 C10 C5 108.79(15) . . ?
C20 C10 C9 106.64(14) . . ?
C20 C10 C5 111.93(15) . . ?
C20 C10 C1 108.11(16) . . ?
C1 C10 C9 113.07(14) . . ?
C1 C10 C5 108.37(15) . . ?
C15 C16 H16 106.6 . . ?
C15 C16 C13 104.12(17) . . ?
C17 C16 C15 112.93(16) . . ?
C17 C16 H16 106.6 . . ?
C17 C16 C13 119.18(17) . . ?
C13 C16 H16 106.6 . . ?
O3 C20 C10 111.47(18) . . ?
O3 C20 H20A 109.3 . . ?
O3 C20 H20B 109.3 . . ?
C10 C20 H20A 109.3 . . ?
C10 C20 H20B 109.3 . . ?
H20A C20 H20B 108.0 . . ?
O1 C11 C9 109.95(15) . . ?
O1 C11 H11 108.7 . . ?
O1 C11 C12 111.09(18) . . ?
C9 C11 H11 108.7 . . ?
C12 C11 C9 109.76(16) . . ?
C12 C11 H11 108.7 . . ?
C15 C8 C9 104.39(14) . . ?
C15 C8 C14 99.73(15) . . ?
C14 C8 C9 112.52(14) . . ?
C7 C8 C9 117.51(14) . . ?

C7 C8 C15 112.25(16) . . ?
C7 C8 C14 108.96(15) . . ?
C8 C14 H14A 111.7 . . ?
C8 C14 H14B 111.7 . . ?
H14A C14 H14B 109.5 . . ?
C13 C14 C8 100.19(15) . . ?
C13 C14 H14A 111.7 . . ?
C13 C14 H14B 111.7 . . ?
O3 C7 C8 120.37(16) . . ?
O5 C7 O3 118.72(18) . . ?
O5 C7 C8 120.78(19) . . ?
C10 C5 H5 107.5 . . ?
C4 C5 C10 117.79(14) . . ?
C4 C5 H5 107.5 . . ?
C6 C5 C10 115.60(19) . . ?
C6 C5 H5 107.5 . . ?
C6 C5 C4 100.23(16) . . ?
C11 C12 H12A 108.8 . . ?
C11 C12 H12B 108.8 . . ?
C11 C12 C13 113.66(15) . . ?
H12A C12 H12B 107.7 . . ?
C13 C12 H12A 108.8 . . ?
C13 C12 H12B 108.8 . . ?
C10 C1 H1A 109.0 . . ?
C10 C1 H1B 109.0 . . ?
H1A C1 H1B 107.8 . . ?
C2 C1 C10 112.78(18) . . ?
C2 C1 H1A 109.0 . . ?
C2 C1 H1B 109.0 . . ?

C16 C17 H17A 109.0 . . ?
C16 C17 H17B 109.0 . . ?
C16 C17 C21 113.11(18) . . ?
H17A C17 H17B 107.8 . . ?
C21 C17 H17A 109.0 . . ?
C21 C17 H17B 109.0 . . ?
C16 C13 H13 110.0 . . ?
C14 C13 C16 101.84(15) . . ?
C14 C13 C12 108.59(18) . . ?
C14 C13 H13 110.0 . . ?
C12 C13 C16 115.93(17) . . ?
C12 C13 H13 110.0 . . ?
C3 C4 C5 109.2(2) . . ?
C18 C4 C5 118.19(17) . . ?
C18 C4 C3 111.2(2) . . ?
C18 C4 C19 109.0(2) . . ?
C19 C4 C5 98.64(18) . . ?
C19 C4 C3 109.8(2) . . ?
C17 C21 H21 107.9 . . ?
C23 C21 C17 111.2(2) . . ?
C23 C21 H21 107.9 . . ?
C22 C21 C17 110.8(2) . . ?
C22 C21 H21 107.9 . . ?
C22 C21 C23 110.9(3) . . ?
C4 C3 H3A 109.1 . . ?
C4 C3 H3B 109.1 . . ?
H3A C3 H3B 107.8 . . ?
C2 C3 C4 112.51(18) . . ?
C2 C3 H3A 109.1 . . ?

C2 C3 H3B 109.1 . . ?
C1 C2 H2A 109.3 . . ?
C1 C2 H2B 109.3 . . ?
C3 C2 C1 111.50(18) . . ?
C3 C2 H2A 109.3 . . ?
C3 C2 H2B 109.3 . . ?
H2A C2 H2B 108.0 . . ?
O4 C6 C5 109.6(2) . . ?
O2 C6 O4 120.4(2) . . ?
O2 C6 C5 130.1(2) . . ?
C4 C18 H18A 109.5 . . ?
C4 C18 H18B 109.5 . . ?
C4 C18 H18C 109.5 . . ?
H18A C18 H18B 109.5 . . ?
H18A C18 H18C 109.5 . . ?
H18B C18 H18C 109.5 . . ?
O4 C19 C4 106.2(3) . . ?
O4 C19 H19A 110.5 . . ?
O4 C19 H19B 110.5 . . ?
C4 C19 H19A 110.5 . . ?
C4 C19 H19B 110.5 . . ?
H19A C19 H19B 108.7 . . ?
C21 C23 H23A 109.5 . . ?
C21 C23 H23B 109.5 . . ?
C21 C23 H23C 109.5 . . ?
H23A C23 H23B 109.5 . . ?
H23A C23 H23C 109.5 . . ?
H23B C23 H23C 109.5 . . ?
C21 C22 H22A 109.5 . . ?

```
  C21 C22 H22B 109.5 . . ?
  C21 C22 H22C 109.5 . . ?
  H22A C22 H22B 109.5 . . ?
  H22A C22 H22C 109.5 . . ?
  H22B C22 H22C 109.5 . . ?

_shelx_res_file
;
       a.res created by SHELXL-2014/7

TITL a_a.res in P2(1)2(1)2(1)
REM Old TITL a in P222 #16
REM SHELXT solution in P2(1)2(1)2(1)
REM R1 0.143, Rweak 0.039, Alpha 0.006, Orientation as input
REM Flack x = 0.081 ( 0.065 ) from Parsons' quotients
REM Formula found by SHELXT: C23 N O5
CELL 1.54178 8.016 13.0903 19.9957 90 90 90
ZERR 4 0.0004 0.0006 0.0009 0 0 0
LATT -1
SYMM 0.5-X,-Y,0.5+Z
SYMM -X,0.5+Y,0.5-Z
SYMM 0.5+X,0.5-Y,-Z
SFAC C H O
UNIT 92 128 24

L.S. 4
PLAN    20
SIZE 0.17 0.15 0.18
TEMP 0
```

```
BOND $H
list 4
fmap 2
ACTA
REM <olex2.extras>
REM <HklSrc "%.\\a.hkl">
REM </olex2.extras>

WGHT    0.100000
FVAR       0.70957
O3    3    0.075257    0.548722    0.676801    11.00000    0.04973    0.02945 =
         0.05393    0.00156   -0.01424    0.00973
O1    3   -0.041258    0.320335    0.787708    11.00000    0.03737    0.04054 =
         0.06633   -0.00328    0.00831   -0.00965
AFIX 147
H1    2   -0.099206    0.268902    0.784820    11.00000   -1.50000
AFIX   0
O6    3    0.534090    0.520102    0.755513    11.00000    0.04694    0.05187 =
         0.04581    0.00879   -0.00302   -0.01316
O5    3    0.224180    0.649988    0.739878    11.00000    0.07555    0.02524 =
         0.06561   -0.00029   -0.01994    0.00313
O4    3    0.394663    0.424473    0.506859    11.00000    0.09393    0.08982 =
         0.03541    0.01623    0.01013    0.00412
O2    3    0.133108    0.466946    0.525927    11.00000    0.08328    0.08958 =
         0.05093    0.02493   -0.01962    0.01004
C9    1    0.225684    0.364347    0.737692    11.00000    0.03374    0.02227 =
         0.02819    0.00013   -0.00123   -0.00109
AFIX  13
H9    2    0.339988    0.337983    0.735597    11.00000   -1.20000
```

```
AFIX    0
C15     1    0.423477    0.484497    0.789136    11.00000    0.04179    0.02796 =
             0.03216   -0.00187   -0.00329   -0.00105
C10     1    0.163697    0.369205    0.664156    11.00000    0.03344    0.02858 =
             0.03198   -0.00056   -0.00529   -0.00075
C16     1    0.441352    0.437424    0.858778    11.00000    0.04434    0.03550 =
             0.02894   -0.00178   -0.00347    0.00250
AFIX   13
H16     2    0.471148    0.493297    0.889132    11.00000   -1.20000
AFIX    0
C20     1    0.018850    0.445061    0.662815    11.00000    0.03783    0.03490 =
             0.04958   -0.00040   -0.01283    0.00337
AFIX   23
H20A    2   -0.033927    0.443220    0.619178    11.00000   -1.20000
H20B    2   -0.063595    0.425139    0.695842    11.00000   -1.20000
AFIX    0
C11     1    0.130647    0.292284    0.785240    11.00000    0.04264    0.02732 =
             0.03887    0.00249    0.00500   -0.00424
AFIX   13
H11     2    0.139199    0.222397    0.768005    11.00000   -1.20000
AFIX    0
C8      1    0.239660    0.474776    0.768912    11.00000    0.03928    0.02358 =
             0.03135   -0.00124   -0.00155    0.00066
C14     1    0.156712    0.482146    0.838841    11.00000    0.04554    0.03569 =
             0.03769   -0.00652    0.00348    0.00554
AFIX   23
H14A    2    0.166773    0.550194    0.857614    11.00000   -1.20000
H14B    2    0.040036    0.462619    0.837410    11.00000   -1.20000
AFIX    0
```

```
C7      1    0.181868    0.563522    0.726680    11.00000    0.04793    0.02444 =
        0.04228   -0.00098   -0.00499    0.00600
C5      1    0.310508    0.404202    0.618807    11.00000    0.04092    0.03288 =
        0.02811    0.00306   -0.00368   -0.00164
AFIX   13
H5      2    0.362679    0.463455    0.640253    11.00000   -1.20000
AFIX    0
C12     1    0.209885    0.295911    0.855151    11.00000    0.05454    0.03777 =
        0.03674    0.00830    0.00639   -0.00420
AFIX   23
H12A    2    0.307605    0.252210    0.855530    11.00000   -1.20000
H12B    2    0.131039    0.268633    0.887327    11.00000   -1.20000
AFIX    0
C1      1    0.099267    0.265675    0.638037    11.00000    0.04292    0.03548 =
        0.04043   -0.00673   -0.00712   -0.00668
AFIX   23
H1A     2    0.016961    0.239131    0.669063    11.00000   -1.20000
H1B     2    0.044502    0.276111    0.595322    11.00000   -1.20000
AFIX    0
C17     1    0.581871    0.359430    0.862971    11.00000    0.04773    0.04707 =
        0.03530    0.00481   -0.00280    0.00965
AFIX   23
H17A    2    0.683346    0.389915    0.845646    11.00000   -1.20000
H17B    2    0.554414    0.301451    0.834839    11.00000   -1.20000
AFIX    0
C13     1    0.261708    0.404422    0.877380    11.00000    0.04807    0.03994 =
        0.02723   -0.00210    0.00462    0.00243
AFIX   13
H13     2    0.243732    0.412517    0.925567    11.00000   -1.20000
```

```
AFIX   0
C4    1    0.450198    0.325868    0.603687    11.00000    0.04438    0.04678 =
         0.03452   -0.00306    0.00328    0.00406
C21   1    0.614096    0.321400    0.934603    11.00000    0.05261    0.05759 =
         0.03848    0.01119   -0.00097    0.00704
AFIX  13
H21   2    0.506038    0.314758    0.957110    11.00000   -1.20000
AFIX   0
C3    1    0.370575    0.224160    0.581303    11.00000    0.06215    0.04548 =
         0.04729   -0.01401    0.00138    0.00946
AFIX  23
H3A   2    0.321084    0.233129    0.537427    11.00000   -1.20000
H3B   2    0.457012    0.172621    0.577551    11.00000   -1.20000
AFIX   0
C2    1    0.238036    0.187028    0.629463    11.00000    0.06182    0.02983 =
         0.05373   -0.00941   -0.00278    0.00069
AFIX  23
H2A   2    0.288735    0.173275    0.672581    11.00000   -1.20000
H2B   2    0.190822    0.123674    0.612886    11.00000   -1.20000
AFIX   0
C6    1    0.262560    0.435728    0.547411    11.00000    0.07099    0.05012 =
         0.03333    0.01019   -0.00662    0.00028
C18   1    0.584131    0.308147    0.656909    11.00000    0.04075    0.07170 =
         0.05226   -0.00252    0.00074    0.01324
AFIX 137
H18A  2    0.615289    0.372346    0.676468    11.00000   -1.50000
H18B  2    0.680174    0.277150    0.636642    11.00000   -1.50000
H18C  2    0.540999    0.263820    0.691026    11.00000   -1.50000
AFIX   0
```

```
C19     1      0.529340    0.379396    0.543497    11.00000    0.06631    0.07622 =
        0.04783    0.00403    0.01879    0.00018
AFIX   23
H19A    2      0.607161    0.431505    0.558203    11.00000    -1.20000
H19B    2      0.588770    0.330579    0.515850    11.00000    -1.20000
AFIX    0
C23     1      0.718842    0.397645    0.973977    11.00000    0.09327    0.08402 =
        0.05538    0.00224    -0.02847    0.00153
AFIX 137
H23A    2      0.828296    0.401820    0.954685    11.00000    -1.50000
H23B    2      0.727472    0.375517    1.019619    11.00000    -1.50000
H23C    2      0.666762    0.463643    0.972439    11.00000    -1.50000
AFIX    0
C22     1      0.696200    0.216914    0.933838    11.00000    0.11204    0.06732 =
        0.06159    0.02092    -0.00811    0.03322
AFIX 137
H22A    2      0.624394    0.169004    0.911643    11.00000    -1.50000
H22B    2      0.715545    0.194714    0.978934    11.00000    -1.50000
H22C    2      0.800588    0.220943    0.910454    11.00000    -1.50000
AFIX    0
HKLF 4

REM   a_a.res in P2(1)2(1)2(1)
REM R1 =   0.0451 for      4267 Fo > 4sig(Fo)   and   0.0479 for all      4524 data
REM      266 parameters refined using         0 restraints

END

WGHT        0.0864        0.1146
```

REM Highest difference peak 0.336, deepest hole -0.262, 1-sigma level 0.053

Q1	1	0.1932	0.3599	0.7016	11.00000	0.05	0.34
Q2	1	0.4296	0.4532	0.8231	11.00000	0.05	0.25
Q3	1	0.2055	0.4800	0.8022	11.00000	0.05	0.24
Q4	1	0.2189	0.4107	0.7490	11.00000	0.05	0.23
Q5	1	0.2795	0.4184	0.5862	11.00000	0.05	0.20
Q6	1	0.6017	0.3369	0.8984	11.00000	0.05	0.16
Q7	1	0.4867	0.4205	0.7148	11.00000	0.05	0.16
Q8	1	0.1684	0.5031	0.4757	11.00000	0.05	0.16
Q9	1	0.0864	0.2976	0.5846	11.00000	0.05	0.15
Q10	1	0.4156	0.4591	0.6939	11.00000	0.05	0.15
Q11	1	0.5640	0.2546	0.7516	11.00000	0.05	0.15
Q12	1	0.4336	0.2521	0.7740	11.00000	0.05	0.15
Q13	1	0.5744	0.3549	0.6915	11.00000	0.05	0.15
Q14	1	-0.0159	0.4835	0.6074	11.00000	0.05	0.15
Q15	1	0.0101	0.4632	0.6076	11.00000	0.05	0.14
Q16	1	0.0456	0.2961	0.9025	11.00000	0.05	0.14
Q17	1	0.2039	0.5134	0.7510	11.00000	0.05	0.14
Q18	1	0.1756	0.3354	0.7613	11.00000	0.05	0.14
Q19	1	0.6155	0.4196	0.7004	11.00000	0.05	0.14
Q20	1	0.4727	0.3860	0.7720	11.00000	0.05	0.14

Crystal Structure Report for Compound **2**

```
data_20190801WYXIRX25_0m_a

_audit_creation_method            'SHELXL-2018/3'
_shelx_SHELXL_version_number      '2018/3'
_chemical_name_systematic         ?
_chemical_name_common             ?
_chemical_melting_point           ?
_chemical_formula_moiety          ?
_chemical_formula_sum
  'C20 H26 O5'
_chemical_formula_weight          346.41

loop_
  _atom_type_symbol
  _atom_type_description
  _atom_type_scat_dispersion_real
  _atom_type_scat_dispersion_imag
  _atom_type_scat_source
  'C'   'C'    0.0033    0.0016
  'International Tables Vol C Tables 4.2.6.8 and 6.1.1.4'
  'H'   'H'    0.0000    0.0000
  'International Tables Vol C Tables 4.2.6.8 and 6.1.1.4'
```

```
 'O'  'O'   0.0106   0.0060
 'International Tables Vol C Tables 4.2.6.8 and 6.1.1.4'

_space_group_crystal_system       monoclinic
_space_group_IT_number            4
_space_group_name_H-M_alt         'P 21'
_space_group_name_Hall            'P 2yb'

_shelx_space_group_comment
;
The symmetry employed for this shelxl refinement is uniquely defined
by the following loop, which should always be used as a source of
symmetry information in preference to the above space-group names.
They are only intended as comments.
;

loop_
 _space_group_symop_operation_xyz
 'x, y, z'
 '-x, y+1/2, -z'

_cell_length_a                    6.4646(2)
_cell_length_b                    21.4751(8)
_cell_length_c                    12.3769(5)
_cell_angle_alpha                 90
_cell_angle_beta                  94.1000(10)
_cell_angle_gamma                 90
_cell_volume                      1713.86(11)
_cell_formula_units_Z             4
```

```
_cell_measurement_temperature          300(2)
_cell_measurement_reflns_used          ?
_cell_measurement_theta_min            ?
_cell_measurement_theta_max            ?

_exptl_crystal_description             ?
_exptl_crystal_colour                  ?
_exptl_crystal_density_meas            ?
_exptl_crystal_density_method          ?
_exptl_crystal_density_diffrn          1.343
_exptl_crystal_F_000                   744
_exptl_transmission_factor_min         ?
_exptl_transmission_factor_max         ?
_exptl_crystal_size_max                ?
_exptl_crystal_size_mid                ?
_exptl_crystal_size_min                ?
_exptl_absorpt_coefficient_mu          0.095
_shelx_estimated_absorpt_T_min         ?
_shelx_estimated_absorpt_T_max         ?
_exptl_absorpt_correction_type         multi-scan
_exptl_absorpt_correction_T_min        0.7060
_exptl_absorpt_correction_T_max        0.7473
_exptl_absorpt_process_details
;
 SADABS 2016/2: Krause, L., Herbst-Irmer, R., Sheldrick G.M. & Stalke D.,
 J. Appl. Cryst. 48 (2015) 3-10
;
_exptl_absorpt_special_details         ?
_diffrn_ambient_temperature            300(2)
```

_diffrn_radiation_wavelength 0.71076
_diffrn_radiation_type MoK\a
_diffrn_source ?
_diffrn_measurement_device_type ?
_diffrn_measurement_method ?
_diffrn_detector_area_resol_mean ?
_diffrn_reflns_number 57561
_diffrn_reflns_av_unetI/netI 0.0378
_diffrn_reflns_av_R_equivalents 0.0358
_diffrn_reflns_limit_h_min -10
_diffrn_reflns_limit_h_max 10
_diffrn_reflns_limit_k_min -36
_diffrn_reflns_limit_k_max 36
_diffrn_reflns_limit_l_min -20
_diffrn_reflns_limit_l_max 20
_diffrn_reflns_theta_min 3.159
_diffrn_reflns_theta_max 37.058
_diffrn_reflns_theta_full 25.243
_diffrn_measured_fraction_theta_max 0.997
_diffrn_measured_fraction_theta_full 0.993
_diffrn_reflns_Laue_measured_fraction_max 0.997
_diffrn_reflns_Laue_measured_fraction_full 0.993
_diffrn_reflns_point_group_measured_fraction_max 0.994
_diffrn_reflns_point_group_measured_fraction_full 0.993
_reflns_number_total 17341
_reflns_number_gt 11735
_reflns_threshold_expression 'I > 2\s(I)'
_reflns_Friedel_coverage 0.954
_reflns_Friedel_fraction_max 0.991

```
_reflns_Friedel_fraction_full          0.993

_reflns_special_details
;
 Reflections were merged by SHELXL according to the crystal
 class for the calculation of statistics and refinement.

 _reflns_Friedel_fraction is defined as the number of unique
 Friedel pairs measured divided by the number that would be
 possible theoretically, ignoring centric projections and
 systematic absences.
;

_computing_data_collection                ?
_computing_cell_refinement                ?
_computing_data_reduction                 ?
_computing_structure_solution          'SHELXT 2014/5 (Sheldrick, 2014)'
_computing_structure_refinement 'SHELXL-2018/3 (Sheldrick, 2018)'
_computing_molecular_graphics          ?
_computing_publication_material      ?
_refine_special_details                   ?
_refine_ls_structure_factor_coef    Fsqd
_refine_ls_matrix_type                    full
_refine_ls_weighting_scheme             calc
_refine_ls_weighting_details
'w=1/[\s^2^(Fo^2^)+(0.0642P)^2^+0.0285P] where P=(Fo^2^+2Fc^2^)/3'
_atom_sites_solution_primary            ?
_atom_sites_solution_secondary        ?
_atom_sites_solution_hydrogens        geom
```

```
_refine_ls_hydrogen_treatment          mixed
_refine_ls_extinction_method           none
_refine_ls_extinction_coef             .
_refine_ls_abs_structure_details
;
  Flack x determined using 4495 quotients [(I+)-(I-)]/[(I+)+(I-)]
  (Parsons, Flack and Wagner, Acta Cryst. B69 (2013) 249-259).
;
_refine_ls_abs_structure_Flack         -0.09(16)
_chemical_absolute_configuration       ?
_refine_ls_number_reflns               17341
_refine_ls_number_parameters           456
_refine_ls_number_restraints           1
_refine_ls_R_factor_all                0.0813
_refine_ls_R_factor_gt                 0.0466
_refine_ls_wR_factor_ref               0.1242
_refine_ls_wR_factor_gt                0.1086
_refine_ls_goodness_of_fit_ref         1.018
_refine_ls_restrained_S_all            1.018
_refine_ls_shift/su_max                0.001
_refine_ls_shift/su_mean               0.000

loop_
  _atom_site_label
  _atom_site_type_symbol
  _atom_site_fract_x
  _atom_site_fract_y
  _atom_site_fract_z
  _atom_site_U_iso_or_equiv
```

_atom_site_adp_type
_atom_site_occupancy
_atom_site_site_symmetry_order
_atom_site_calc_flag
_atom_site_refinement_flags_posn
_atom_site_refinement_flags_adp
_atom_site_refinement_flags_occupancy
_atom_site_disorder_assembly
_atom_site_disorder_group
O1 O -0.0372(2) 0.38817(6) 0.23593(9) 0.0462(3) Uani 1 1 d . . . . .
C1 C -0.1750(3) 0.39256(9) -0.05779(14) 0.0410(3) Uani 1 1 d . . . . .
H1A H -0.090981 0.386557 -0.118817 0.049 Uiso 1 1 calc R U . . .
H1AB H -0.285972 0.362070 -0.063575 0.049 Uiso 1 1 calc R U . . .
O2 O -0.2733(2) 0.31873(7) 0.16099(12) 0.0498(3) Uani 1 1 d . . . . .
C2 C -0.2691(4) 0.45773(11) -0.06413(17) 0.0557(5) Uani 1 1 d . . . . .
H2A H -0.350489 0.462602 -0.132550 0.067 Uiso 1 1 calc R U . . .
H2AB H -0.361049 0.463054 -0.006216 0.067 Uiso 1 1 calc R U . . .
O3 O -0.3011(2) 0.21873(7) 0.15082(13) 0.0516(3) Uani 1 1 d . . . . .
C3 C -0.1017(4) 0.50690(9) -0.05463(16) 0.0541(5) Uani 1 1 d . . . . .
H3A H -0.166421 0.547642 -0.059884 0.065 Uiso 1 1 calc R U . . .
H3AB H -0.015280 0.502590 -0.115097 0.065 Uiso 1 1 calc R U . . .
O4 O 0.2536(2) 0.21498(7) 0.17380(9) 0.0432(3) Uani 1 1 d . . . . .
H4 H 0.369(5) 0.2186(14) 0.1543(8) 0.065 Uiso 1 1 calc R U . . .
C4 C 0.0360(3) 0.50349(7) 0.05091(14) 0.0404(3) Uani 1 1 d . . . . .
O5 O 0.0324(3) 0.49479(7) 0.23976(11) 0.0506(3) Uani 1 1 d . . . . .
C5 C 0.1025(2) 0.43620(7) 0.08362(12) 0.0308(3) Uani 1 1 d . . . . .
H5 H 0.243347 0.428261 0.062575 0.037 Uiso 1 1 calc R U . . .
C6 C 0.1006(3) 0.43555(8) 0.20852(13) 0.0383(3) Uani 1 1 d . . . . .
H6 H 0.240154 0.427226 0.241601 0.046 Uiso 1 1 calc R U . . .

C7 C -0.2060(2) 0.26404(8) 0.12566(12) 0.0353(3) Uani 1 1 d . . . . .
C8 C -0.0306(2) 0.26095(7) 0.05123(11) 0.0286(2) Uani 1 1 d . . . . .
C9 C 0.09464(19) 0.32160(6) 0.04707(10) 0.0249(2) Uani 1 1 d . . . . .
H9 H 0.180382 0.322867 0.115607 0.030 Uiso 1 1 calc R U . . .
C10 C -0.0409(2) 0.38108(7) 0.04721(11) 0.0276(2) Uani 1 1 d . . . . .
C12 C 0.1897(3) 0.28024(8) -0.14346(13) 0.0388(3) Uani 1 1 d . . . . .
H12A H 0.097553 0.304162 -0.192905 0.047 Uiso 1 1 calc R U . . .
H12B H 0.312983 0.270231 -0.180236 0.047 Uiso 1 1 calc R U . . .
C11 C 0.2507(2) 0.31966(8) -0.04219(12) 0.0326(3) Uani 1 1 d . . . . .
H11A H 0.274063 0.362048 -0.065503 0.039 Uiso 1 1 calc R U . . .
H11B H 0.381726 0.304038 -0.009951 0.039 Uiso 1 1 calc R U . . .
C13 C 0.0818(3) 0.22008(8) -0.11324(13) 0.0380(3) Uani 1 1 d . . . . .
H13 H 0.054951 0.192676 -0.175989 0.046 Uiso 1 1 calc R U . . .
C14 C -0.1180(3) 0.23752(8) -0.06143(13) 0.0365(3) Uani 1 1 d . . . . .
H14A H -0.207779 0.201707 -0.055198 0.044 Uiso 1 1 calc R U . . .
H14B H -0.193201 0.270067 -0.101886 0.044 Uiso 1 1 calc R U . . .
C15 C 0.1138(2) 0.20458(7) 0.08336(12) 0.0335(3) Uani 1 1 d . . . . .
H15 H 0.024119 0.169921 0.101626 0.040 Uiso 1 1 calc R U . . .
C16 C 0.2072(3) 0.18767(7) -0.02171(13) 0.0353(3) Uani 1 1 d . . . . .
C17 C 0.3726(3) 0.15253(9) -0.03023(17) 0.0454(4) Uani 1 1 d . . . . .
H17A H 0.422869 0.145669 -0.097758 0.055 Uiso 1 1 calc R U . . .
H17B H 0.438881 0.134623 0.031296 0.055 Uiso 1 1 calc R U . . .
C18 C 0.2303(4) 0.54336(9) 0.0434(2) 0.0628(6) Uani 1 1 d . . . . .
H18A H 0.309812 0.527670 -0.013332 0.094 Uiso 1 1 calc R U . . .
H18B H 0.312569 0.541789 0.111033 0.094 Uiso 1 1 calc R U . . .
H18C H 0.190630 0.585658 0.027653 0.094 Uiso 1 1 calc R U . . .
C19 C -0.0761(3) 0.52468(9) 0.14936(15) 0.0461(4) Uani 1 1 d . . . . .
H19A H -0.069790 0.569606 0.157061 0.055 Uiso 1 1 calc R U . . .
H19B H -0.220381 0.511907 0.142672 0.055 Uiso 1 1 calc R U . . .

C20 C -0.1733(3) 0.37657(8) 0.14455(14) 0.0379(3) Uani 1 1 d . . . . .
H20 H -0.278376 0.409527 0.138831 0.046 Uiso 1 1 calc R U . . .
C1_1 C 0.7001(3) 0.68442(10) 0.55496(16) 0.0464(4) Uani 1 1 d . . . . .
H1A_1 H 0.668846 0.676647 0.629261 0.056 Uiso 1 1 calc R U . . .
H1AB_1 H 0.795605 0.652342 0.534666 0.056 Uiso 1 1 calc R U . . .
O1_1 O 0.3708(3) 0.69379(8) 0.30023(10) 0.0570(4) Uani 1 1 d . . . . .
C2_1 C 0.8066(3) 0.74738(13) 0.5494(2) 0.0568(5) Uani 1 1 d . . . . .
H2A_1 H 0.927276 0.748086 0.600740 0.068 Uiso 1 1 calc R U . . .
H2AB_1 H 0.853788 0.753261 0.477494 0.068 Uiso 1 1 calc R U . . .
O2_1 O 0.5851(3) 0.61058(8) 0.32462(11) 0.0557(4) Uani 1 1 d . . . . .
C3_1 C 0.6635(4) 0.80004(11) 0.57461(18) 0.0546(5) Uani 1 1 d . . . . .
H3A_1 H 0.737833 0.839080 0.570172 0.065 Uiso 1 1 calc R U . . .
H3AB_1 H 0.624623 0.795456 0.648452 0.065 Uiso 1 1 calc R U . . .
O3_1 O 0.5223(3) 0.51119(8) 0.31487(13) 0.0594(4) Uani 1 1 d . . . . .
C4_1 C 0.4666(3) 0.80312(9) 0.49886(14) 0.0435(4) Uani 1 1 d . . . . .
O4_1 O 0.0431(2) 0.53472(8) 0.45117(11) 0.0490(3) Uani 1 1 d . . . . .
H4_1 H 0.012091 0.523513 0.388769 0.074 Uiso 1 1 calc R U . . .
C5_1 C 0.3649(3) 0.73897(8) 0.47610(12) 0.0353(3) Uani 1 1 d . . . . .
H5_1 H 0.248497 0.733842 0.521912 0.042 Uiso 1 1 calc R U . . .
O5_1 O 0.3371(3) 0.80133(8) 0.31643(13) 0.0665(5) Uani 1 1 d . . . . .
C6_1 C 0.2805(3) 0.74293(10) 0.35606(15) 0.0476(4) Uani 1 1 d . . . . .
H6_1 H 0.129218 0.738681 0.350595 0.057 Uiso 1 1 calc R U . . .
C7_1 C 0.5066(3) 0.55795(9) 0.36600(14) 0.0423(4) Uani 1 1 d . . . . .
C8_1 C 0.4229(2) 0.56001(8) 0.47695(12) 0.0354(3) Uani 1 1 d . . . . .
C9_1 C 0.3526(2) 0.62514(7) 0.51032(11) 0.0323(3) Uani 1 1 d . . . . .
H9_1 H 0.223002 0.632712 0.466263 0.039 Uiso 1 1 calc R U . . .
C10_1 C 0.4985(2) 0.67894(8) 0.48164(11) 0.0339(3) Uani 1 1 d . . . . .
C11_1 C 0.2909(3) 0.62560(9) 0.62927(12) 0.0423(4) Uani 1 1 d . . . . .
H11A_1 H 0.304470 0.667851 0.656576 0.051 Uiso 1 1 calc R U . . .

H11B_1 H 0.145668 0.614233 0.629322 0.051 Uiso 1 1 calc R U . . .
C12_1 C 0.4157(4) 0.58248(10) 0.70800(14) 0.0546(5) Uani 1 1 d . . . . .
H12A_1 H 0.340347 0.576144 0.772110 0.065 Uiso 1 1 calc R U . . .
H12B_1 H 0.547357 0.601920 0.730306 0.065 Uiso 1 1 calc R U . . .
C13_1 C 0.4552(4) 0.51932(10) 0.65529(16) 0.0549(5) Uani 1 1 d . . . . .
H13_1 H 0.522711 0.490025 0.707300 0.066 Uiso 1 1 calc R U . . .
C14_1 C 0.5853(3) 0.53033(10) 0.55913(17) 0.0494(4) Uani 1 1 d . . . . .
H14A_1 H 0.638753 0.491596 0.531977 0.059 Uiso 1 1 calc R U . . .
H14B_1 H 0.699696 0.558510 0.577517 0.059 Uiso 1 1 calc R U . . .
C15_1 C 0.2421(3) 0.51231(8) 0.48458(14) 0.0406(3) Uani 1 1 d . . . . .
H15_1 H 0.271123 0.475826 0.440559 0.049 Uiso 1 1 calc R U . . .
C16_1 C 0.2574(4) 0.49306(9) 0.60296(15) 0.0492(4) Uani 1 1 d . . . . .
C17_1 C 0.1174(5) 0.45913(13) 0.6492(2) 0.0674(6) Uani 1 1 d . . . . .
H17A_1 H 0.136544 0.449129 0.722293 0.081 Uiso 1 1 calc R U . . .
H17B_1 H -0.000236 0.445289 0.608484 0.081 Uiso 1 1 calc R U . . .
C18_1 C 0.3092(4) 0.84631(10) 0.5474(2) 0.0580(5) Uani 1 1 d . . . . .
H18A_1 H 0.371423 0.886321 0.561767 0.087 Uiso 1 1 calc R U . . .
H18B_1 H 0.267351 0.828738 0.613745 0.087 Uiso 1 1 calc R U . . .
H18C_1 H 0.190073 0.850948 0.497117 0.087 Uiso 1 1 calc R U . . .
C19_1 C 0.5051(4) 0.82555(11) 0.38483(17) 0.0587(5) Uani 1 1 d . . . . .
H19A_1 H 0.506970 0.870682 0.381894 0.070 Uiso 1 1 calc R U . . .
H19B_1 H 0.636539 0.809921 0.362920 0.070 Uiso 1 1 calc R U . . .
C20_1 C 0.5459(3) 0.67154(9) 0.36283(14) 0.0445(4) Uani 1 1 d . . . . .
H20_1 H 0.664683 0.698035 0.349252 0.053 Uiso 1 1 calc R U . . .

loop_
 _atom_site_aniso_label
 _atom_site_aniso_U_11
 _atom_site_aniso_U_22

_atom_site_aniso_U_33
_atom_site_aniso_U_23
_atom_site_aniso_U_13
_atom_site_aniso_U_12
O1 0.0659(8) 0.0484(7) 0.0250(5) 0.0008(5) 0.0082(5) -0.0041(6)
C1 0.0442(8) 0.0452(8) 0.0320(7) 0.0002(6) -0.0084(6) 0.0072(7)
O2 0.0441(6) 0.0519(7) 0.0568(7) -0.0016(6) 0.0277(6) -0.0086(6)
C2 0.0616(12) 0.0618(12) 0.0413(9) 0.0043(8) -0.0125(8) 0.0271(10)
O3 0.0450(7) 0.0547(8) 0.0566(8) 0.0131(6) 0.0130(6) -0.0158(6)
C3 0.0854(15) 0.0413(9) 0.0360(8) 0.0085(7) 0.0068(9) 0.0219(10)
O4 0.0426(6) 0.0576(7) 0.0287(5) 0.0052(5) -0.0025(4) 0.0077(6)
C4 0.0539(9) 0.0282(7) 0.0401(8) 0.0023(6) 0.0107(7) 0.0086(6)
O5 0.0730(9) 0.0434(7) 0.0345(6) -0.0101(5) -0.0033(6) 0.0138(6)
C5 0.0344(6) 0.0269(6) 0.0314(6) 0.0001(5) 0.0043(5) 0.0017(5)
C6 0.0474(8) 0.0353(7) 0.0311(7) -0.0052(6) -0.0032(6) 0.0045(6)
C7 0.0299(6) 0.0450(8) 0.0308(6) 0.0074(6) 0.0021(5) -0.0082(6)
C8 0.0282(6) 0.0320(6) 0.0256(5) 0.0033(5) 0.0012(4) -0.0053(5)
C9 0.0248(5) 0.0282(5) 0.0217(5) 0.0020(4) 0.0021(4) -0.0025(5)
C10 0.0280(6) 0.0314(6) 0.0238(5) 0.0023(5) 0.0031(4) 0.0004(5)
C12 0.0514(9) 0.0388(8) 0.0275(6) 0.0010(6) 0.0110(6) 0.0009(7)
C11 0.0339(6) 0.0321(6) 0.0332(6) 0.0000(5) 0.0114(5) -0.0041(6)
C13 0.0506(9) 0.0353(7) 0.0276(6) -0.0049(5) -0.0009(6) -0.0031(6)
C14 0.0396(7) 0.0371(7) 0.0317(6) 0.0000(6) -0.0061(5) -0.0082(6)
C15 0.0376(7) 0.0309(6) 0.0318(6) 0.0057(5) 0.0010(5) -0.0039(5)
C16 0.0426(7) 0.0290(6) 0.0339(7) -0.0020(5) -0.0001(6) -0.0043(6)
C17 0.0499(9) 0.0395(8) 0.0467(9) -0.0067(7) 0.0022(7) 0.0019(7)
C18 0.0719(14) 0.0301(8) 0.0893(17) 0.0047(9) 0.0258(13) -0.0019(8)
C19 0.0601(10) 0.0397(8) 0.0387(8) -0.0035(7) 0.0047(7) 0.0159(8)
C20 0.0382(7) 0.0411(8) 0.0360(7) 0.0005(6) 0.0132(6) 0.0012(6)

C1_1 0.0370(7) 0.0583(10) 0.0429(9) -0.0033(8) -0.0028(7) 0.0019(8)
O1_1 0.0813(10) 0.0616(9) 0.0270(5) -0.0050(5) -0.0038(6) 0.0097(8)
C2_1 0.0396(9) 0.0700(13) 0.0600(12) -0.0074(10) -0.0017(8) -0.0118(9)
O2_1 0.0744(10) 0.0574(8) 0.0387(6) -0.0084(6) 0.0263(7) 0.0059(7)
C3_1 0.0594(11) 0.0585(11) 0.0460(10) -0.0110(9) 0.0049(9) -0.0164(9)
O3_1 0.0626(9) 0.0637(9) 0.0543(8) -0.0260(7) 0.0202(7) 0.0015(7)
C4_1 0.0544(9) 0.0429(9) 0.0345(7) -0.0023(6) 0.0117(7) -0.0063(7)
O4_1 0.0419(6) 0.0657(9) 0.0392(6) -0.0133(6) 0.0009(5) -0.0004(6)
C5_1 0.0383(7) 0.0401(7) 0.0277(6) 0.0001(6) 0.0038(5) 0.0009(6)
O5_1 0.1024(13) 0.0534(8) 0.0421(7) 0.0134(6) -0.0068(8) -0.0029(9)
C6_1 0.0580(10) 0.0494(10) 0.0344(8) 0.0041(7) -0.0050(7) 0.0030(8)
C7_1 0.0374(7) 0.0559(10) 0.0340(7) -0.0123(7) 0.0056(6) 0.0065(7)
C8_1 0.0370(7) 0.0413(8) 0.0278(6) -0.0061(6) 0.0010(5) 0.0069(6)
C9_1 0.0357(7) 0.0395(7) 0.0220(5) -0.0034(5) 0.0034(5) 0.0044(6)
C10_1 0.0353(6) 0.0438(8) 0.0229(5) -0.0035(5) 0.0040(5) 0.0023(6)
C11_1 0.0594(10) 0.0424(8) 0.0261(6) -0.0038(6) 0.0110(7) 0.0023(7)
C12_1 0.0833(14) 0.0537(11) 0.0257(7) 0.0005(7) -0.0030(8) -0.0016(10)
C13_1 0.0765(14) 0.0490(10) 0.0370(8) 0.0058(8) -0.0114(9) 0.0083(10)
C14_1 0.0511(9) 0.0478(10) 0.0475(9) -0.0025(8) -0.0091(8) 0.0145(8)
C15_1 0.0482(8) 0.0414(8) 0.0324(7) -0.0091(6) 0.0042(6) 0.0020(7)
C16_1 0.0700(12) 0.0390(8) 0.0389(8) -0.0010(7) 0.0064(8) 0.0045(8)
C17_1 0.0896(18) 0.0575(13) 0.0569(13) 0.0054(10) 0.0180(12) -0.0035(12)
C18_1 0.0784(15) 0.0396(9) 0.0582(12) -0.0044(8) 0.0203(11) 0.0031(9)
C19_1 0.0841(15) 0.0526(11) 0.0411(9) 0.0036(9) 0.0154(9) -0.0147(11)
C20_1 0.0542(9) 0.0507(10) 0.0300(7) -0.0021(7) 0.0119(7) 0.0003(8)

_geom_special_details
;
All esds (except the esd in the dihedral angle between two l.s. planes)

```
  are estimated using the full covariance matrix.  The cell esds are taken
  into account individually in the estimation of esds in distances, angles
  and torsion angles; correlations between esds in cell parameters are only
  used when they are defined by crystal symmetry.  An approximate (isotropic)
  treatment of cell esds is used for estimating esds involving l.s. planes.
;

loop_
  _geom_bond_atom_site_label_1
  _geom_bond_atom_site_label_2
  _geom_bond_distance
  _geom_bond_site_symmetry_2
  _geom_bond_publ_flag
O1 C20 1.405(2) . ?
O1 C6 1.410(2) . ?
C1 C2 1.526(3) . ?
C1 C10 1.530(2) . ?
C1 H1A 0.9700 . ?
C1 H1AB 0.9700 . ?
O2 C7 1.337(2) . ?
O2 C20 1.422(2) . ?
C2 C3 1.511(4) . ?
C2 H2A 0.9700 . ?
C2 H2AB 0.9700 . ?
O3 C7 1.204(2) . ?
C3 C4 1.529(3) . ?
C3 H3A 0.9700 . ?
C3 H3AB 0.9700 . ?
O4 C15 1.405(2) . ?
```

O4 H4 0.80(3) . ?
C4 C18 1.529(3) . ?
C4 C19 1.531(2) . ?
C4 C5 1.553(2) . ?
O5 C6 1.409(2) . ?
O5 C19 1.430(2) . ?
C5 C6 1.547(2) . ?
C5 C10 1.550(2) . ?
C5 H5 0.9800 . ?
C6 H6 0.9800 . ?
C7 C8 1.513(2) . ?
C8 C9 1.5365(19) . ?
C8 C14 1.550(2) . ?
C8 C15 1.563(2) . ?
C9 C11 1.5490(18) . ?
C9 C10 1.5490(19) . ?
C9 H9 0.9800 . ?
C10 C20 1.530(2) . ?
C12 C13 1.527(2) . ?
C12 C11 1.540(2) . ?
C12 H12A 0.9700 . ?
C12 H12B 0.9700 . ?
C11 H11A 0.9700 . ?
C11 H11B 0.9700 . ?
C13 C16 1.514(2) . ?
C13 C14 1.529(2) . ?
C13 H13 0.9800 . ?
C14 H14A 0.9700 . ?
C14 H14B 0.9700 . ?

C15 C16 1.516(2) . ?
C15 H15 0.9800 . ?
C16 C17 1.319(3) . ?
C17 H17A 0.9300 . ?
C17 H17B 0.9300 . ?
C18 H18A 0.9600 . ?
C18 H18B 0.9600 . ?
C18 H18C 0.9600 . ?
C19 H19A 0.9700 . ?
C19 H19B 0.9700 . ?
C20 H20 0.9800 . ?
C1_1 C2_1 1.521(3) . ?
C1_1 C10_1 1.538(2) . ?
C1_1 H1A_1 0.9700 . ?
C1_1 H1AB_1 0.9700 . ?
O1_1 C20_1 1.409(3) . ?
O1_1 C6_1 1.410(3) . ?
C2_1 C3_1 1.508(3) . ?
C2_1 H2A_1 0.9700 . ?
C2_1 H2AB_1 0.9700 . ?
O2_1 C7_1 1.354(3) . ?
O2_1 C20_1 1.421(2) . ?
C3_1 C4_1 1.527(3) . ?
C3_1 H3A_1 0.9700 . ?
C3_1 H3AB_1 0.9700 . ?
O3_1 C7_1 1.195(2) . ?
C4_1 C19_1 1.528(3) . ?
C4_1 C18_1 1.531(3) . ?
C4_1 C5_1 1.544(2) . ?

O4_1 C15_1 1.408(2) . ?
O4_1 H4_1 0.8200 . ?
C5_1 C6_1 1.548(2) . ?
C5_1 C10_1 1.551(2) . ?
C5_1 H5_1 0.9800 . ?
O5_1 C6_1 1.405(3) . ?
O5_1 C19_1 1.427(3) . ?
C6_1 H6_1 0.9800 . ?
C7_1 C8_1 1.512(2) . ?
C8_1 C9_1 1.536(2) . ?
C8_1 C14_1 1.546(2) . ?
C8_1 C15_1 1.562(3) . ?
C9_1 C10_1 1.549(2) . ?
C9_1 C11_1 1.553(2) . ?
C9_1 H9_1 0.9800 . ?
C10_1 C20_1 1.532(2) . ?
C11_1 C12_1 1.532(3) . ?
C11_1 H11A_1 0.9700 . ?
C11_1 H11B_1 0.9700 . ?
C12_1 C13_1 1.534(3) . ?
C12_1 H12A_1 0.9700 . ?
C12_1 H12B_1 0.9700 . ?
C13_1 C16_1 1.502(3) . ?
C13_1 C14_1 1.524(3) . ?
C13_1 H13_1 0.9800 . ?
C14_1 H14A_1 0.9700 . ?
C14_1 H14B_1 0.9700 . ?
C15_1 C16_1 1.519(3) . ?
C15_1 H15_1 0.9800 . ?

C16_1 C17_1 1.323(3) . ?
C17_1 H17A_1 0.9300 . ?
C17_1 H17B_1 0.9300 . ?
C18_1 H18A_1 0.9600 . ?
C18_1 H18B_1 0.9600 . ?
C18_1 H18C_1 0.9600 . ?
C19_1 H19A_1 0.9700 . ?
C19_1 H19B_1 0.9700 . ?
C20_1 H20_1 0.9800 . ?

loop_
  _geom_angle_atom_site_label_1
  _geom_angle_atom_site_label_2
  _geom_angle_atom_site_label_3
  _geom_angle
  _geom_angle_site_symmetry_1
  _geom_angle_site_symmetry_3
  _geom_angle_publ_flag
C20 O1 C6 107.73(12) . . ?
C2 C1 C10 113.02(14) . . ?
C2 C1 H1A 109.0 . . ?
C10 C1 H1A 109.0 . . ?
C2 C1 H1AB 109.0 . . ?
C10 C1 H1AB 109.0 . . ?
H1A C1 H1AB 107.8 . . ?
C7 O2 C20 123.96(11) . . ?
C3 C2 C1 110.87(17) . . ?
C3 C2 H2A 109.5 . . ?
C1 C2 H2A 109.5 . . ?

C3 C2 H2AB 109.5 . . ?
C1 C2 H2AB 109.5 . . ?
H2A C2 H2AB 108.1 . . ?
C2 C3 C4 113.78(16) . . ?
C2 C3 H3A 108.8 . . ?
C4 C3 H3A 108.8 . . ?
C2 C3 H3AB 108.8 . . ?
C4 C3 H3AB 108.8 . . ?
H3A C3 H3AB 107.7 . . ?
C15 O4 H4 109.5 . . ?
C18 C4 C3 110.60(17) . . ?
C18 C4 C19 108.66(17) . . ?
C3 C4 C19 112.71(16) . . ?
C18 C4 C5 108.88(15) . . ?
C3 C4 C5 113.61(14) . . ?
C19 C4 C5 101.95(13) . . ?
C6 O5 C19 109.65(12) . . ?
C6 C5 C10 103.68(12) . . ?
C6 C5 C4 104.30(12) . . ?
C10 C5 C4 119.08(13) . . ?
C6 C5 H5 109.7 . . ?
C10 C5 H5 109.7 . . ?
C4 C5 H5 109.7 . . ?
O5 C6 O1 111.71(14) . . ?
O5 C6 C5 106.91(13) . . ?
O1 C6 C5 107.28(12) . . ?
O5 C6 H6 110.3 . . ?
O1 C6 H6 110.3 . . ?
C5 C6 H6 110.3 . . ?

O3 C7 O2 116.17(14) . . ?
O3 C7 C8 122.76(16) . . ?
O2 C7 C8 120.90(13) . . ?
C7 C8 C9 113.76(12) . . ?
C7 C8 C14 108.74(12) . . ?
C9 C8 C14 113.82(11) . . ?
C7 C8 C15 109.83(12) . . ?
C9 C8 C15 111.01(11) . . ?
C14 C8 C15 98.64(12) . . ?
C8 C9 C11 111.88(11) . . ?
C8 C9 C10 113.55(10) . . ?
C11 C9 C10 114.86(11) . . ?
C8 C9 H9 105.1 . . ?
C11 C9 H9 105.1 . . ?
C10 C9 H9 105.1 . . ?
C1 C10 C20 111.36(13) . . ?
C1 C10 C9 114.71(12) . . ?
C20 C10 C9 107.28(11) . . ?
C1 C10 C5 114.50(12) . . ?
C20 C10 C5 100.13(12) . . ?
C9 C10 C5 107.66(11) . . ?
C13 C12 C11 111.13(12) . . ?
C13 C12 H12A 109.4 . . ?
C11 C12 H12A 109.4 . . ?
C13 C12 H12B 109.4 . . ?
C11 C12 H12B 109.4 . . ?
H12A C12 H12B 108.0 . . ?
C12 C11 C9 116.94(12) . . ?
C12 C11 H11A 108.1 . . ?

C9 C11 H11A 108.1 . . ?
C12 C11 H11B 108.1 . . ?
C9 C11 H11B 108.1 . . ?
H11A C11 H11B 107.3 . . ?
C16 C13 C12 110.05(14) . . ?
C16 C13 C14 102.81(13) . . ?
C12 C13 C14 108.03(13) . . ?
C16 C13 H13 111.8 . . ?
C12 C13 H13 111.8 . . ?
C14 C13 H13 111.8 . . ?
C13 C14 C8 101.08(12) . . ?
C13 C14 H14A 111.6 . . ?
C8 C14 H14A 111.6 . . ?
C13 C14 H14B 111.6 . . ?
C8 C14 H14B 111.6 . . ?
H14A C14 H14B 109.4 . . ?
O4 C15 C16 116.65(13) . . ?
O4 C15 C8 114.72(13) . . ?
C16 C15 C8 103.56(12) . . ?
O4 C15 H15 107.1 . . ?
C16 C15 H15 107.1 . . ?
C8 C15 H15 107.1 . . ?
C17 C16 C13 126.63(16) . . ?
C17 C16 C15 125.46(16) . . ?
C13 C16 C15 107.88(13) . . ?
C16 C17 H17A 120.0 . . ?
C16 C17 H17B 120.0 . . ?
H17A C17 H17B 120.0 . . ?
C4 C18 H18A 109.5 . . ?

C4 C18 H18B 109.5 . . ?
H18A C18 H18B 109.5 . . ?
C4 C18 H18C 109.5 . . ?
H18A C18 H18C 109.5 . . ?
H18B C18 H18C 109.5 . . ?
O5 C19 C4 104.82(14) . . ?
O5 C19 H19A 110.8 . . ?
C4 C19 H19A 110.8 . . ?
O5 C19 H19B 110.8 . . ?
C4 C19 H19B 110.8 . . ?
H19A C19 H19B 108.9 . . ?
O1 C20 O2 107.75(14) . . ?
O1 C20 C10 105.63(13) . . ?
O2 C20 C10 116.98(13) . . ?
O1 C20 H20 108.7 . . ?
O2 C20 H20 108.7 . . ?
C10 C20 H20 108.7 . . ?
C2_1 C1_1 C10_1 114.11(17) . . ?
C2_1 C1_1 H1A_1 108.7 . . ?
C10_1 C1_1 H1A_1 108.7 . . ?
C2_1 C1_1 H1AB_1 108.7 . . ?
C10_1 C1_1 H1AB_1 108.7 . . ?
H1A_1 C1_1 H1AB_1 107.6 . . ?
C20_1 O1_1 C6_1 109.18(14) . . ?
C3_1 C2_1 C1_1 111.77(17) . . ?
C3_1 C2_1 H2A_1 109.3 . . ?
C1_1 C2_1 H2A_1 109.3 . . ?
C3_1 C2_1 H2AB_1 109.3 . . ?
C1_1 C2_1 H2AB_1 109.3 . . ?

H2A_1 C2_1 H2AB_1 107.9 . . ?
C7_1 O2_1 C20_1 124.23(13) . . ?
C2_1 C3_1 C4_1 113.73(17) . . ?
C2_1 C3_1 H3A_1 108.8 . . ?
C4_1 C3_1 H3A_1 108.8 . . ?
C2_1 C3_1 H3AB_1 108.8 . . ?
C4_1 C3_1 H3AB_1 108.8 . . ?
H3A_1 C3_1 H3AB_1 107.7 . . ?
C3_1 C4_1 C19_1 113.39(18) . . ?
C3_1 C4_1 C18_1 109.47(17) . . ?
C19_1 C4_1 C18_1 109.15(19) . . ?
C3_1 C4_1 C5_1 113.51(16) . . ?
C19_1 C4_1 C5_1 102.02(15) . . ?
C18_1 C4_1 C5_1 109.00(16) . . ?
C15_1 O4_1 H4_1 109.5 . . ?
C4_1 C5_1 C6_1 104.02(14) . . ?
C4_1 C5_1 C10_1 120.32(14) . . ?
C6_1 C5_1 C10_1 104.18(13) . . ?
C4_1 C5_1 H5_1 109.2 . . ?
C6_1 C5_1 H5_1 109.2 . . ?
C10_1 C5_1 H5_1 109.2 . . ?
C6_1 O5_1 C19_1 108.93(16) . . ?
O5_1 C6_1 O1_1 111.70(18) . . ?
O5_1 C6_1 C5_1 107.56(16) . . ?
O1_1 C6_1 C5_1 107.43(15) . . ?
O5_1 C6_1 H6_1 110.0 . . ?
O1_1 C6_1 H6_1 110.0 . . ?
C5_1 C6_1 H6_1 110.0 . . ?
O3_1 C7_1 O2_1 116.92(16) . . ?

O3_1 C7_1 C8_1 123.70(19) . . ?
O2_1 C7_1 C8_1 119.14(15) . . ?
C7_1 C8_1 C9_1 113.75(14) . . ?
C7_1 C8_1 C14_1 108.36(14) . . ?
C9_1 C8_1 C14_1 113.48(13) . . ?
C7_1 C8_1 C15_1 110.71(13) . . ?
C9_1 C8_1 C15_1 110.11(13) . . ?
C14_1 C8_1 C15_1 99.53(15) . . ?
C8_1 C9_1 C10_1 114.89(12) . . ?
C8_1 C9_1 C11_1 111.21(13) . . ?
C10_1 C9_1 C11_1 114.49(13) . . ?
C8_1 C9_1 H9_1 105.0 . . ?
C10_1 C9_1 H9_1 105.0 . . ?
C11_1 C9_1 H9_1 105.0 . . ?
C20_1 C10_1 C1_1 110.81(14) . . ?
C20_1 C10_1 C9_1 108.02(13) . . ?
C1_1 C10_1 C9_1 115.21(14) . . ?
C20_1 C10_1 C5_1 101.12(13) . . ?
C1_1 C10_1 C5_1 114.11(14) . . ?
C9_1 C10_1 C5_1 106.49(12) . . ?
C12_1 C11_1 C9_1 116.10(15) . . ?
C12_1 C11_1 H11A_1 108.3 . . ?
C9_1 C11_1 H11A_1 108.3 . . ?
C12_1 C11_1 H11B_1 108.3 . . ?
C9_1 C11_1 H11B_1 108.3 . . ?
H11A_1 C11_1 H11B_1 107.4 . . ?
C11_1 C12_1 C13_1 111.10(15) . . ?
C11_1 C12_1 H12A_1 109.4 . . ?
C13_1 C12_1 H12A_1 109.4 . . ?

C11_1 C12_1 H12B_1 109.4 . . ?
C13_1 C12_1 H12B_1 109.4 . . ?
H12A_1 C12_1 H12B_1 108.0 . . ?
C16_1 C13_1 C14_1 102.82(16) . . ?
C16_1 C13_1 C12_1 110.67(19) . . ?
C14_1 C13_1 C12_1 108.22(18) . . ?
C16_1 C13_1 H13_1 111.6 . . ?
C14_1 C13_1 H13_1 111.6 . . ?
C12_1 C13_1 H13_1 111.6 . . ?
C13_1 C14_1 C8_1 101.02(15) . . ?
C13_1 C14_1 H14A_1 111.6 . . ?
C8_1 C14_1 H14A_1 111.6 . . ?
C13_1 C14_1 H14B_1 111.6 . . ?
C8_1 C14_1 H14B_1 111.6 . . ?
H14A_1 C14_1 H14B_1 109.4 . . ?
O4_1 C15_1 C16_1 111.80(15) . . ?
O4_1 C15_1 C8_1 115.47(15) . . ?
C16_1 C15_1 C8_1 103.86(15) . . ?
O4_1 C15_1 H15_1 108.5 . . ?
C16_1 C15_1 H15_1 108.5 . . ?
C8_1 C15_1 H15_1 108.5 . . ?
C17_1 C16_1 C13_1 127.5(2) . . ?
C17_1 C16_1 C15_1 124.6(2) . . ?
C13_1 C16_1 C15_1 107.97(17) . . ?
C16_1 C17_1 H17A_1 120.0 . . ?
C16_1 C17_1 H17B_1 120.0 . . ?
H17A_1 C17_1 H17B_1 120.0 . . ?
C4_1 C18_1 H18A_1 109.5 . . ?
C4_1 C18_1 H18B_1 109.5 . . ?

H18A_1 C18_1 H18B_1 109.5 . . ?
C4_1 C18_1 H18C_1 109.5 . . ?
H18A_1 C18_1 H18C_1 109.5 . . ?
H18B_1 C18_1 H18C_1 109.5 . . ?
O5_1 C19_1 C4_1 105.41(18) . . ?
O5_1 C19_1 H19A_1 110.7 . . ?
C4_1 C19_1 H19A_1 110.7 . . ?
O5_1 C19_1 H19B_1 110.7 . . ?
C4_1 C19_1 H19B_1 110.7 . . ?
H19A_1 C19_1 H19B_1 108.8 . . ?
O1_1 C20_1 O2_1 106.55(16) . . ?
O1_1 C20_1 C10_1 106.56(15) . . ?
O2_1 C20_1 C10_1 117.90(15) . . ?
O1_1 C20_1 H20_1 108.5 . . ?
O2_1 C20_1 H20_1 108.5 . . ?
C10_1 C20_1 H20_1 108.5 . . ?

_refine_diff_density_max 0.281
_refine_diff_density_min -0.143
_refine_diff_density_rms 0.035

_shelx_res_file
;
TITL 20190801WYXIRX25_0m_a.res in P2(1)
    20190801WYXIRX25_0m_a.res
    created by SHELXL-2018/3 at 11:28:29 on 06-Feb-2022
REM Old TITL Integration of 20190801WYXIRX25
REM SHELXT solution in P2(1)
REM R1 0.133, Rweak 0.038, Alpha 0.007, Orientation as input

```
REM Flack x =    0.022 ( 0.123 ) from Parsons' quotients
REM Formula found by SHELXT:    C39 O11
CELL    0.71076      6.4646    21.4751    12.3769      90.000      94.100      90.000
ZERR        4.000      0.0002      0.0008      0.0005       0.000       0.001       0.000
LATT -1
SYMM -X, 1/2+Y, -Z
SFAC C H O
UNIT 80 104 20
TEMP 26.870
ACTA
L.S. 10
BOND $H
LIST 4
FMAP 2
PLAN 5
WGHT        0.064200        0.028500
FVAR             1.33315
O1        3     -0.037212       0.388166       0.235929       11.00000       0.06588       0.04843 =
                0.02499       0.00080       0.00819      -0.00410
C1        1     -0.175015       0.392555      -0.057790       11.00000       0.04423       0.04524 =
                0.03198       0.00020      -0.00836       0.00720
AFIX    23
H1A      2     -0.090981       0.386557      -0.118817       11.00000      -1.20000
H1AB    2     -0.285972       0.362070      -0.063575       11.00000      -1.20000
AFIX     0
O2        3     -0.273343       0.318733       0.160985       11.00000       0.04410       0.05185 =
                0.05683      -0.00159       0.02770      -0.00858
C2        1     -0.269137       0.457726      -0.064134       11.00000       0.06164       0.06179 =
                0.04129       0.00433      -0.01247       0.02708
```

```
AFIX   23
H2A     2    -0.350489    0.462602   -0.132550    11.00000   -1.20000
H2AB    2    -0.361049    0.463054   -0.006216    11.00000   -1.20000
AFIX    0
O3      3    -0.301143    0.218732    0.150816    11.00000    0.04497    0.05467 =
         0.05660    0.01311    0.01298   -0.01577
C3      1    -0.101731    0.506896   -0.054625    11.00000    0.08544    0.04131 =
         0.03599    0.00852    0.00681    0.02190
AFIX   23
H3A     2    -0.166421    0.547642   -0.059884    11.00000   -1.20000
H3AB    2    -0.015280    0.502590   -0.115097    11.00000   -1.20000
AFIX    0
O4      3     0.253600    0.214978    0.173796    11.00000    0.04261    0.05755 =
         0.02874    0.00524   -0.00248    0.00769
AFIX 148
H4      2     0.368507    0.218597    0.154343    11.00000   -1.50000
AFIX    0
C4      1     0.035956    0.503492    0.050915    11.00000    0.05390    0.02821 =
         0.04008    0.00232    0.01073    0.00855
O5      3     0.032427    0.494786    0.239757    11.00000    0.07295    0.04336 =
         0.03446   -0.01011   -0.00328    0.01384
C5      1     0.102452    0.436195    0.083618    11.00000    0.03444    0.02694 =
         0.03144    0.00008    0.00434    0.00175
AFIX   13
H5      2     0.243347    0.428261    0.062575    11.00000   -1.20000
AFIX    0
C6      1     0.100620    0.435546    0.208522    11.00000    0.04745    0.03534 =
         0.03110   -0.00519   -0.00324    0.00450
AFIX   13
```

```
H6      2     0.240154    0.427226    0.241601    11.00000   -1.20000
AFIX    0
C7      1    -0.205983    0.264040    0.125661    11.00000    0.02992    0.04503 =
             0.03082    0.00737    0.00214   -0.00819
C8      1    -0.030596    0.260948    0.051233    11.00000    0.02819    0.03201 =
             0.02558    0.00330    0.00116   -0.00531
C9      1     0.094637    0.321604    0.047066    11.00000    0.02475    0.02818 =
             0.02172    0.00202    0.00210   -0.00250
AFIX   13
H9      2     0.180382    0.322867    0.115607    11.00000   -1.20000
AFIX    0
C10     1    -0.040906    0.381084    0.047209    11.00000    0.02795    0.03137 =
             0.02375    0.00228    0.00305    0.00035
C12     1     0.189658    0.280239   -0.143458    11.00000    0.05137    0.03884 =
             0.02749    0.00101    0.01103    0.00085
AFIX   23
H12A    2     0.097553    0.304162   -0.192905    11.00000   -1.20000
H12B    2     0.312983    0.270231   -0.180236    11.00000   -1.20000
AFIX    0
C11     1     0.250702    0.319660   -0.042192    11.00000    0.03392    0.03207 =
             0.03317   -0.00001    0.01137   -0.00411
AFIX   23
H11A    2     0.274063    0.362048   -0.065503    11.00000   -1.20000
H11B    2     0.381726    0.304038   -0.009951    11.00000   -1.20000
AFIX    0
C13     1     0.081844    0.220077   -0.113237    11.00000    0.05058    0.03531 =
             0.02760   -0.00489   -0.00087   -0.00310
AFIX   13
H13     2     0.054951    0.192676   -0.175989    11.00000   -1.20000
```

```
AFIX   0
C14    1   -0.117977    0.237521   -0.061430    11.00000    0.03959    0.03706 =
           0.03170   -0.00003   -0.00608   -0.00818
AFIX  23
H14A   2   -0.207779    0.201707   -0.055198    11.00000   -1.20000
H14B   2   -0.193201    0.270067   -0.101886    11.00000   -1.20000
AFIX   0
C15    1    0.113840    0.204584    0.083360    11.00000    0.03765    0.03090 =
           0.03184    0.00574    0.00096   -0.00387
AFIX  13
H15    2    0.024119    0.169921    0.101626    11.00000   -1.20000
AFIX   0
C16    1    0.207195    0.187675   -0.021712    11.00000    0.04261    0.02899 =
           0.03387   -0.00204   -0.00011   -0.00430
C17    1    0.372571    0.152535   -0.030228    11.00000    0.04989    0.03952 =
           0.04672   -0.00673    0.00224    0.00190
AFIX  93
H17A   2    0.422869    0.145669   -0.097758    11.00000   -1.20000
H17B   2    0.438881    0.134623    0.031296    11.00000   -1.20000
AFIX   0
C18    1    0.230321    0.543361    0.043365    11.00000    0.07192    0.03012 =
           0.08927    0.00473    0.02578   -0.00193
AFIX 137
H18A   2    0.309812    0.527670   -0.013332    11.00000   -1.50000
H18B   2    0.312569    0.541789    0.111033    11.00000   -1.50000
H18C   2    0.190630    0.585658    0.027653    11.00000   -1.50000
AFIX   0
C19    1   -0.076113    0.524682    0.149362    11.00000    0.06006    0.03968 =
           0.03875   -0.00355    0.00473    0.01590
```

```
AFIX   23
H19A   2    -0.069790    0.569606    0.157061    11.00000   -1.20000
H19B   2    -0.220381    0.511907    0.142672    11.00000   -1.20000
AFIX    0
C20    1    -0.173300    0.376572    0.144555    11.00000    0.03818    0.04115 =
            0.03600    0.00054    0.01322    0.00116
AFIX   13
H20    2    -0.278376    0.409527    0.138831    11.00000   -1.20000
AFIX    0
RESI 1
C1     1     0.700096    0.684416    0.554960    11.00000    0.03701    0.05834 =
            0.04293   -0.00331   -0.00280    0.00195
AFIX   23
H1A    2     0.668846    0.676647    0.629261    11.00000   -1.20000
H1AB   2     0.795605    0.652342    0.534666    11.00000   -1.20000
AFIX    0
O1     3     0.370810    0.693792    0.300227    11.00000    0.08127    0.06164 =
            0.02695   -0.00501   -0.00379    0.00968
C2     1     0.806612    0.747382    0.549448    11.00000    0.03959    0.07003 =
            0.05998   -0.00738   -0.00173   -0.01176
AFIX   23
H2A    2     0.927276    0.748086    0.600740    11.00000   -1.20000
H2AB   2     0.853788    0.753261    0.477494    11.00000   -1.20000
AFIX    0
O2     3     0.585064    0.610576    0.324616    11.00000    0.07438    0.05738 =
            0.03865   -0.00837    0.02631    0.00593
C3     1     0.663453    0.800040    0.574605    11.00000    0.05936    0.05852 =
            0.04597   -0.01102    0.00494   -0.01635
AFIX   23
```

```
H3A    2   0.737833   0.839080   0.570172   11.00000   -1.20000
H3AB   2   0.624623   0.795456   0.648452   11.00000   -1.20000
AFIX   0
O3     3   0.522306   0.511195   0.314874   11.00000    0.06257    0.06372 =
        0.05434   -0.02599    0.02019    0.00154
C4     1   0.466624   0.803119   0.498855   11.00000    0.05440    0.04289 =
        0.03454   -0.00228    0.01173   -0.00625
O4     3   0.043128   0.534717   0.451166   11.00000    0.04185    0.06566 =
        0.03923   -0.01333    0.00095   -0.00036
AFIX 147
H4     2   0.012091   0.523513   0.388769   11.00000   -1.50000
AFIX   0
C5     1   0.364852   0.738968   0.476100   11.00000    0.03833    0.04008 =
        0.02771    0.00013    0.00381    0.00090
AFIX  13
H5     2   0.248497   0.733842   0.521912   11.00000   -1.20000
AFIX   0
O5     3   0.337095   0.801334   0.316435   11.00000    0.10239    0.05339 =
        0.04213    0.01337   -0.00683   -0.00294
C6     1   0.280542   0.742929   0.356064   11.00000    0.05797    0.04936 =
        0.03441    0.00409   -0.00500    0.00297
AFIX  13
H6     2   0.129218   0.738681   0.350595   11.00000   -1.20000
AFIX   0
C7     1   0.506634   0.557948   0.366002   11.00000    0.03740    0.05592 =
        0.03402   -0.01234    0.00559    0.00647
C8     1   0.422908   0.560010   0.476952   11.00000    0.03697    0.04135 =
        0.02777   -0.00614    0.00102    0.00689
C9     1   0.352602   0.625139   0.510320   11.00000    0.03565    0.03946 =
```

```
               0.02200   -0.00343    0.00340    0.00445
AFIX  13
H9    2    0.223002    0.632712    0.466263    11.00000   -1.20000
AFIX   0
C10   1    0.498508    0.678943    0.481640    11.00000    0.03532    0.04382 =
               0.02288   -0.00352    0.00404    0.00229
C11   1    0.290878    0.625602    0.629269    11.00000    0.05943    0.04241 =
               0.02612   -0.00375    0.01104    0.00225
AFIX  23
H11A  2    0.304470    0.667851    0.656576    11.00000   -1.20000
H11B  2    0.145668    0.614233    0.629322    11.00000   -1.20000
AFIX   0
C12   1    0.415740    0.582477    0.708003    11.00000    0.08334    0.05367 =
               0.02571    0.00048   -0.00300   -0.00159
AFIX  23
H12A  2    0.340347    0.576144    0.772110    11.00000   -1.20000
H12B  2    0.547357    0.601920    0.730306    11.00000   -1.20000
AFIX   0
C13   1    0.455201    0.519317    0.655289    11.00000    0.07647    0.04901 =
               0.03696    0.00577   -0.01138    0.00834
AFIX  13
H13   2    0.522711    0.490025    0.707300    11.00000   -1.20000
AFIX   0
C14   1    0.585293    0.530330    0.559128    11.00000    0.05110    0.04783 =
               0.04755   -0.00248   -0.00914    0.01446
AFIX  23
H14A  2    0.638753    0.491596    0.531977    11.00000   -1.20000
H14B  2    0.699696    0.558510    0.577517    11.00000   -1.20000
AFIX   0
```

```
C15     1     0.242132     0.512307     0.484583     11.00000     0.04823     0.04135 =
          0.03241    -0.00909     0.00424     0.00203
AFIX   13
H15     2     0.271123     0.475826     0.440559     11.00000    -1.20000
AFIX    0
C16     1     0.257372     0.493062     0.602956     11.00000     0.07001     0.03897 =
          0.03895    -0.00100     0.00645     0.00449
C17     1     0.117354     0.459132     0.649155     11.00000     0.08958     0.05754 =
          0.05689     0.00536     0.01800    -0.00348
AFIX   93
H17A    2     0.136544     0.449129     0.722293     11.00000    -1.20000
H17B    2    -0.000236     0.445289     0.608484     11.00000    -1.20000
AFIX    0
C18     1     0.309185     0.846309     0.547398     11.00000     0.07843     0.03962 =
          0.05816    -0.00437     0.02034     0.00306
AFIX 137
H18A    2     0.371423     0.886321     0.561767     11.00000    -1.50000
H18B    2     0.267351     0.828738     0.613745     11.00000    -1.50000
H18C    2     0.190073     0.850948     0.497117     11.00000    -1.50000
AFIX    0
C19     1     0.505123     0.825551     0.384834     11.00000     0.08410     0.05259 =
          0.04106     0.00357     0.01539    -0.01471
AFIX   23
H19A    2     0.506970     0.870682     0.381894     11.00000    -1.20000
H19B    2     0.636539     0.809921     0.362920     11.00000    -1.20000
AFIX    0
C20     1     0.545876     0.671537     0.362833     11.00000     0.05416     0.05071 =
          0.02995    -0.00213     0.01190     0.00028
AFIX   13
```

```
H20     2     0.664683     0.698035     0.349252     11.00000     -1.20000
RESI 0
AFIX     0
HKLF 4




REM     20190801WYXIRX25_0m_a.res in P2(1)
REM wR2 = 0.124171, GooF = S = 1.01847, Restrained GooF = 1.01844 for all data
REM R1 = 0.046581 for 11735 Fo > 4sig(Fo) and 0.081328 for all 17341 data
REM 456 parameters refined using 1 restraints
```
